# The role of exercise intensity in motor and non-motor symptoms of Parkinson's disease: mechanisms, clinical evidence, and personalized exercise prescription parameters

**DOI:** 10.3389/fnagi.2026.1831208

**Published:** 2026-06-29

**Authors:** Zhiwei Zhang, Yang Zhou

**Affiliations:** 1 Guilin University of Aerospace Technology, Guilin, China; 2 Guangxi Science and Technology Normal University, Laibin, China

**Keywords:** exercise intensity, motor symptoms, neuroplasticity, non-motor symptoms, Parkinson's disease, personalized exercise prescription

## Abstract

Parkinson's disease (PD) is the second most common neurodegenerative disorder, featuring progressive dopaminergic neuronal loss, Lewy body (LB) pathology, and a broad spectrum of motor and non-motor symptoms. Although dopaminergic pharmacotherapy remains the cornerstone of symptomatic treatment, long-term medication use is frequently complicated by motor fluctuations, dyskinesia, and limited efficacy for several non-motor symptoms. Exercise has therefore gained increasing attention as a non-pharmacological intervention for PD. This narrative review summarizes current evidence on how exercise intensity influences motor symptoms, non-motor symptoms, and putative biological mechanisms in PD. Low-intensity exercise appears suitable for improving tolerance, mobility, and adherence, particularly in frail or advanced patients. Moderate-intensity exercise may provide a favorable balance between safety and functional benefit, whereas high-intensity aerobic exercise (AE) and high-intensity interval training may improve cardiorespiratory fitness and selected motor outcomes in carefully screened and supervised patients. For non-motor symptoms, exercise effects appear domain-specific: Aerobic and socially engaging exercise may be more relevant for mood symptoms; cognitively demanding and dual-task exercise may benefit executive function; and lower-intensity or timing-adjusted protocols may be preferable for patients with fatigue, sleep disturbance, or autonomic dysfunction. Potential mechanisms include modulation of neurotrophic signaling, neuroplasticity, neuroinflammation, oxidative stress, and cerebral perfusion; however, much of the mechanistic evidence remains indirect or derived from preclinical models. Current clinical evidence is limited by heterogeneous definitions of exercise intensity, small samples, short intervention periods, limited long-term follow-up, and underrepresentation of advanced PD. Future studies should standardize intensity reporting, assess clinically meaningful changes rather than statistical significance alone, and integrate clinical outcomes with validated biomarkers to support individualized exercise prescription.

## 1 Introduction

Parkinson's disease (PD), the second most common neurodegenerative disorder after Alzheimer's disease (AD), has progressively become a major worldwide public health issue. As the aging population in China increases dramatically, PD has turned into a major threat to elderly health. Its key pathological features include progressive degeneration of dopaminergic neurons in the substantia nigra pars compacta and the formation of intracellular Lewy bodies (LBs; Kouli et al., 2018). These pathological alterations disrupt basal ganglia circuitry and contribute to a broad spectrum of progressively worsening motor and non-motor symptoms. These symptoms can severely compromise patients' daily functioning while causing an overwhelming burden on family caregiving and healthcare systems (Pasquini et al., 2018).

From a symptomatic perspective, the motor manifestations of PD are characterized by the “parkinsonian syndrome,” including four hallmark features: resting tremor, bradykinesia, rigidity, and postural instability. Resting tremor generally originates unilaterally in the hand, producing a characteristic “pill-rolling” motion. Its development is closely associated with specific neuroimaging modifications, making it a critical indicator for early diagnosis (Pasquini et al., 2018). Bradykinesia is characterized by delayed initiation of voluntary movements and a gradual decrease in the speed and amplitude of repetitive actions. Patients generally display “facial masking” and “micrographia” (small handwriting). Literature on manual tracking tasks suggests that PD patients generally generate much clumsier “submovements,” implying significant impairments in the brain's motor planning and execution capabilities (Noy et al., 2025). Muscle rigidity presents as “lead-pipe” or “gear-like” resistance during passive joint movement, tightly linked to abnormal regulation of reflexes by supraspinal centers. Elevated conduction efficiency in the Type II heteronymous pathway of the lower limbs in newly diagnosed PD patients has been considered a key mechanism (Simonetta Moreau et al., 2002). Postural instability frequently occurs in the middle to late stages of the disease. Hindered balance reflexes make patients prone to falls from minor external forces or simple turning movements, functioning as an important feature for distinguishing PD from other parkinsonian syndromes (Shin et al., 2022).

Non-motor symptoms are also highly prevalent in PD. Several symptoms may appear earlier than motor symptoms, and their influence on patients' quality of life is comparable to that of motor impairments. Among these, cognitive dysfunction may progress from mild cognitive impairment (MCI) to PD dementia, mainly impacting executive function, attention, and visuospatial abilities, while early memory storage capacity remains relatively preserved. Personalized interventions are needed (Zhang et al., 2020). Depression and anxiety are the most prevalent emotional disturbances among PD non-motor symptoms. A cross-sectional study reported a significant association between depressive symptoms and decreased quality of life in PD patients, notably impacting emotional wellbeing (Sujith et al., 2024). Moreover, anxiety–depression states display an adverse association with psychological resilience, stressing the crucial significance of psychological interventions (Cai et al., 2025). Notably, sleep disturbances occur in different forms, including insomnia, fragmented sleep, and extreme daytime sleepiness. Among these, rapid eye movement sleep behavior disorder (RBD) is a characteristic feature. PD patients with RBD generally display a heavier burden of non-motor symptoms, such as depression and fatigue (Neikrug et al., 2014). It is noteworthy that autonomic dysfunction can impact nearly all autonomically innervated organs, whose symptoms include orthostatic hypotension, constipation, urinary urgency, and others. Constipation is generally one of the earliest prodromal symptoms of PD, yet effective pharmacological treatment options remain limited (Pfeiffer, 2020; Kulshreshtha et al., 2020).

Current PD treatment focuses on dopaminergic replacement therapy (e.g., levodopa), which can mitigate motor symptoms but may drive long-term complications, such as levodopa-driven dyskinesia (LID; Aguiar et al., 2013). The management of non-motor symptoms is more complex. No clear disease-modifying therapy has been established for PD-related cognitive decline, and autonomic dysfunction often responds incompletely to current pharmacological treatments (Zhang et al., 2020; Kulshreshtha et al., 2020). Previous studies have reported that exercise may improve gait, balance, mood, and quality of life, and may influence biological pathways related to neuroplasticity, oxidative stress, and neuroinflammation (Petzinger et al., 2013). Mitochondrial dysfunction has also been highlighted as an important exercise-responsive mechanism in PD, with exercise potentially improving mitochondrial biogenesis, mitophagy, respiratory chain plasticity, and oxidative stress regulation (Kong et al., 2024b). Recent evidence further suggests that exercise may act as a multi-organ regulatory stimulus linking skeletal muscle metabolism, hepatic energy homeostasis, and brain neuroimmune signaling, thereby providing a broader biological framework for exercise-related benefits in aging-related neurodegeneration and PD (Kong et al., 2025). For example, high-intensity interval training (HIIT) can increase hippocampal neurogenesis and spatial memory in rats through upregulation of brain-derived neurotrophic factor (BDNF; Okamoto et al., 2021); Tai Chi exercise can decrease fall risk by 50% in PD patients, with effects enduring for up to 3 months (Li et al., 2012); and early-stage PD patients undergoing 6 months of endurance training show significant enhancements in peak oxygen uptake (VO_2_peak) and motor function, regardless of autonomic nervous system status (Griffith et al., 2025).

Nevertheless, clinical translation of exercise protocols in PD interventions faces major bottlenecks: (1) The “dose–response” correlation between exercise parameters (intensity, type, duration, frequency) and therapeutic effectiveness remains unclear. For example, while moderate-intensity aerobic exercise (AE; 70 −80% HRmax) can promote cerebral blood flow and BDNF expression (Maass et al., 2015), comparative evidence on its effectiveness vs. HIIT for decreasing motor symptoms in advanced PD is lacking (Schenkman et al., 2018); (2) Significant individual variability exists, with disease stage, genetic background (e.g., BDNF Val66Met polymorphism), and comorbidities impacting exercise response. Early-stage patients may tolerate high-intensity training to increase neuroplasticity (Salvatore et al., 2022), while late-stage patients need low-impact intervention approaches to decrease fall risk (Demonceau et al., 2017); (3) The molecular processes underlying exercise-induced neuroprotective mechanisms, particularly regarding PD-specific α-synuclein pathology, remain incompletely understood. Notably, regulatory pathways involving oxidative stress, neuroinflammation, and synaptic plasticity need further assessment (Salvatore et al., 2022; Demonceau et al., 2017); (4) The absence of biomarkers (e.g., serum BDNF and brain connectivity imaging metrics) to quantify exercise effectiveness and predict response remains a limitation (Kaagman et al., 2024).

This review synthesizes current evidence on exercise intensity in PD, focusing on three questions: (1) how different exercise intensities influence motor symptoms; (2) how exercise intensity may affect non-motor symptoms; and (3) how intensity, modality, disease stage, safety, and patient-specific goals can be integrated into a personalized exercise prescription. Rather than treating exercise as a uniform intervention, this review emphasizes the heterogeneity of exercise intensity definitions, the distinction between statistical and clinically meaningful outcomes, and the need to separate established clinical effects from plausible but incompletely validated biological mechanisms.

## 2 Methods for literature identification and evidence synthesis

This article was designed as a narrative review supported by a structured literature search. Relevant studies were identified through searches of PubMed/MEDLINE, Web of Science, Scopus, Embase, and the Cochrane Library from database inception to March 1, 2026. Search terms included combinations of “Parkinson's disease,” “exercise,” “physical activity,” “exercise intensity,” “aerobic exercise,” “resistance training,” “strength training,” “balance training,” “Tai Chi,” “high-intensity interval training,” “motor symptoms,” “non-motor symptoms,” “cognition,” “depression,” “sleep,” “BDNF,” “neuroplasticity,” “neuroinflammation,” “oxidative stress,” and “biomarker.” We included randomized controlled trials, non-randomized clinical studies, systematic reviews, meta-analyses, and mechanistic studies that addressed exercise intensity, exercise modality, motor outcomes, non-motor outcomes, or biological mechanisms relevant to PD. Animal and preclinical studies were considered only for mechanistic interpretation and were not used as preliminary evidence of clinical efficacy in humans. Given the narrative nature of this review, formal risk-of-bias tools such as RoB 2, ROBINS-I, or AMSTAR 2 were not systematically applied. Methodological quality was, however, considered qualitatively, with priority given to randomized controlled trials, systematic reviews, meta-analyses, and PD-specific human studies (Table 1).

**Table 1:** Hierarchical classification of evidence sources and their interpretive roles in this narrative review.

Study type	Evidence level	Use in this review
PD randomized controlled trials	Highest direct clinical evidence	Used to support clinical effects
PD systematic reviews/meta-analyses	High-level synthesis	Used for overall trends
PD observational studies	Supportive evidence	Used cautiously
Human non-PD studies	Indirect evidence	Used only for mechanistic context
Animal studies	Preclinical mechanistic evidence	Not used as direct clinical proof

Studies were excluded if they did not involve PD or PD-relevant mechanisms, did not include exercise or physical activity interventions, lacked relevance to exercise intensity or symptom outcomes, or were not available in English. Because the included literature was heterogeneous in study design, intervention protocol, intensity definition, outcome measures, and follow-up duration, no quantitative meta-analysis was performed. Formal risk-of-bias scoring was not conducted; however, evidence was interpreted according to study design, population characteristics, sample size, intervention duration, outcome relevance, and whether the evidence was derived from human clinical studies, indirect human biomarker studies, or animal models. The reporting approach was informed by principles from PRISMA-ScR and SANRA for transparent literature identification and narrative evidence synthesis (Tricco et al., 2018; Baethge et al., 2019).

## 3 Findings

### 3.1 Overview of PD

PD is a common neurodegenerative disorder, second only to AD, defined pathologically by progressive loss of dopaminergic neurons in the substantia nigra pars compacta and the formation of intracellular LBs (Kouli et al., 2018). This neuronal degeneration leads to dysfunction in basal ganglia circuits, resulting in a range of complex motor and non-motor symptoms (Mcgregor and Nelson, 2019). As the disease progresses, these symptoms substantially impair patients' activities of daily living and place a considerable burden on families and society.

#### 3.1.1 Motor symptoms

The key motor symptoms of PD, generally termed “parkinsonism,” include bradykinesia, tremor, rigidity, and postural instability. The most typical tremor is a resting tremor, presenting when the limb is completely relaxed and unsupported, and is reduced or absent during voluntary movement. This tremor generally begins unilaterally in the hand, exhibiting a “pill-rolling” motion, and can increasingly spread to the ipsilateral lower limb, contralateral limbs, jaw, and lips (Pasquini et al., 2018). Previous studies have reported that tremor development is linked to specific neuroimaging modifications, functioning as a significant early disease marker (Pasquini et al., 2018). Notably, bradykinesia, a key symptom of PD, means slowness in movement initiation and a decrease in the speed and amplitude of repetitive movements. Patients may demonstrate decreased facial expression (hypomimia), monotonous and soft speech, micrographia, and a shuffling gait with short steps (Noy et al., 2025). Previous studies on manual tracking tasks suggest that PD patients generate more non-fluid “submovements” in comparison to healthy peers, implying impairments in brain planning and execution of smooth, coordinated movements (Noy et al., 2025). Rigidity refers to enhanced resistance during passive joint movement, resembling bending a lead pipe; if tremor is present, a “cogwheel” resistance may be felt. Mechanisms of rigidity involve abnormal reflex regulation by the central nervous system above the spinal level. Studies show that the conduction efficiency of Group II heteronymous pathways in the rigid lower limbs of newly diagnosed PD patients is enhanced (Simonetta Moreau et al., 2002). Direct evaluations of trunk and hip torque also indicate axial hypertonia in PD patients (Wright et al., 2007). Postural instability increases susceptibility to falls, particularly during turning or external perturbations. This is a major trigger of disability and decreased quality of life. Postural instability is a key clinical feature distinguishing PD from other parkinsonian syndromes (Shin et al., 2022).

#### 3.1.2 Non-motor symptoms

Non-motor symptoms are highly prevalent in PD, sometimes preceding typical motor symptoms, and their influence on quality of life is comparable to that of motor disability. Progression from MCI to PD dementia is common. Cognitive dysfunction can mainly impact executive functions (e.g., planning, decision-making, and working memory), attention, and visuospatial abilities, while early memory storage remains relatively preserved. Managing cognitive dysfunction requires comprehensive evaluation and personalized approaches (Zhang et al., 2020). Depression and anxiety are the most common emotional issues in PD patients. A cross-sectional study reported a significant association between depressive symptoms and decreased quality of life in PD patients, notably in the emotional wellbeing domain (Sujith et al., 2024). Moreover, anxiety–depression status is closely linked to patients' levels of psychological resilience, with lower resilience linked to a higher risk of mood disorders, stressing the significance of psychological intervention (Cai et al., 2025). Notably, sleep disturbances in PD are various, including insomnia (difficulty falling or staying asleep), sleep fragmentation, and extreme daytime sleepiness (Claassen and Kutscher, 2011). RBD is notably characteristic, where patients act out their dreams, such as shouting or punching. Evidence has shown that PD patients with RBD generally display a more severe burden of non-motor symptoms, particularly depression, sleep disturbances, and fatigue (Neikrug et al., 2014). It is noteworthy that autonomic dysfunction in PD can impact nearly all organs supplied by the autonomic nervous system. Clinical manifestations include cardiovascular issues (e.g., orthostatic hypotension), gastrointestinal problems (e.g., constipation, gastroparesis, and dysphagia), urinary symptoms (e.g., urgency and frequency), and thermoregulatory abnormalities (e.g., extreme sweating; Pfeiffer, 2020). Gastrointestinal symptoms, notably constipation, are generally among the earliest prodromal signs of PD, possibly involving both central and enteric nervous systems (Kim and Sung, 2015). Currently, effective pharmacological treatments for autonomic dysfunction remain limited, which poses a challenge for clinical management and underscores the need for novel therapeutic approaches (Kulshreshtha et al., 2020).

### 3.2 . Effects of exercise intensity on PD motor symptoms

#### 3.2.1 Exercise parameters and effects

##### 3.2.1.1 Exercise intensity: low, moderate, and high

Exercise intervention is a key approach in the non-pharmacological management of PD, and the correlation between its intensity and the enhancement of motor symptoms has become a focus of clinical and basic research. Low-intensity, moderate-intensity, and high-intensity exercise each have distinct features in alleviating motor symptoms in PD, with different mechanisms and effects regarding neuroplasticity, bradykinesia, and tremor, warranting careful synthesis and review. In this review, exercise intensity is interpreted using a pragmatic multidimensional framework rather than a single universal threshold. For aerobic exercise, intensity was considered according to reported heart rate indices, percentage of maximal oxygen uptake or oxygen uptake reserve, metabolic equivalents, treadmill speed or workload, and rating of perceived exertion. For resistance or strength training (ST), intensity was interpreted according to percentage of one-repetition maximum, number of repetitions, sets, rest intervals, and perceived exertion. Low intensity generally refers to activities performed with a light perceived effort or low cardiovascular load; moderate intensity refers to exercise that produces a clear but tolerable cardiovascular or muscular challenge; and high intensity refers to vigorous continuous exercise or interval-based protocols requiring careful screening and supervision. In PD, heart rate-based prescription may be less reliable in some patients because of autonomic dysfunction, chronotropic impairment, cardiovascular comorbidities, or medication effects. Therefore, clinical tolerance, fall risk, fatigue, orthostatic symptoms, and cognitive status should be considered together with physiological indices when prescribing intensity (American College of Sports Medicine, 2009; Milani et al., 2024). Respiratory frequency may also be considered as a supplementary monitoring variable alongside heart rate and rating of perceived exertion, particularly when autonomic dysfunction, medication effects, or chronotropic impairment reduce the reliability of heart rate-based intensity prescription (Di Paco and Cannataro, 2025).

Low-intensity exercise, such as walking or low-load AE, shows good tolerability and symptom relief in the early stages of PD. This type of exercise may help alleviate bradykinesia and limb rigidity by enhancing blood circulation, preventing muscle atrophy, and improving mood. For example, (Chang et al. 2018) conducted an 8-week progressive low-intensity cycling training (3 times/week, 30 min/session, intensity 40–50% max HR), demonstrating significant improvement in Unified PD Rating Scale (UPDRS)-III scores in early PD patients and showing advantages for motor capacity, notably bradykinesia. In addition, (Van Der Kolk et al. 2019) further indicated sustained benefits through lightly supervised low-intensity AE (3 times/week, 30–45 min/session, intensity 60–70% max HR), demonstrating long-term beneficial effects on motor capacity despite a slower onset. These findings confirm the foundational rehabilitative significance of low-intensity exercise for early PD patients. Nevertheless, its efficiency in mid- to late-stage patients or those with significant functional limitations remains unknown, particularly whether the magnitude of enhancement is sufficient for high symptom loads (Shulman et al., 2013). Hence, future studies should focus on the impact of moderate- and high-intensity exercise in advanced symptom control and their neural processes.

Moderate-intensity exercise (e.g., AE at 70–80% max HR) demonstrates more comprehensive advantages in alleviating PD motor symptoms and enhancing neuroplasticity. A study by (Maass et al. 2015) reported that this intensity can increase cerebral oxygen supply, promote hemodynamic enhancements, and drive vascular plasticity modifications in the hippocampus, leading to significant reductions in bradykinesia and tremor. This process may involve the upregulation of neurotrophic factors, such as BDNF. (Cuccurullo et al. 2024) further stressed that AE can improve motor–cognitive integration by enhancing cerebral blood flow and BDNF expression. This reveals that moderate-intensity exercise, while maintaining safety, has multi-target potential for regulating neural function. Nevertheless, robust evidence for its dose–effect correlation and adaptability across different PD subtypes is still insufficient. Future studies should determine its optimal intensity scope and mechanisms of action on specific neural pathways.

High-intensity exercise, particularly HIIT, may improve cardiorespiratory fitness and selected motor outcomes in PD when applied to carefully screened patients under supervision. Preclinical studies suggest that high-intensity exercise can modulate BDNF signaling, oxidative stress, and inflammatory pathways (Kong et al., 2024b; Freitas et al., 2018). However, these findings should be interpreted as mechanistic support rather than direct proof of clinical efficacy in humans. In clinical settings, the applicability of HIIT is limited by disease stage, fall risk, cardiovascular status, autonomic dysfunction, fatigue, cognitive impairment, and long-term adherence. Therefore, high-intensity protocols should not be generalized to all PD patients and should be prescribed only after individualized risk–benefit assessment (Griffith et al., 2025; Harvey et al., 2019).

A critical issue in interpreting exercise trials is the distinction between statistical significance and clinically meaningful improvement. Where available, changes in motor outcomes should be interpreted against established minimum clinically important difference (MCID) thresholds. For example, an improvement of approximately 3.25 points on the Movement Disorder Society-Unified Parkinson's Disease Rating Scale (MDS-UPDRS) Part III has been proposed as a minimal clinically important improvement, whereas a worsening of approximately 4.63 points may indicate clinically meaningful deterioration (Horváth et al., 2015). However, many exercise studies report statistically significant changes in UPDRS or MDS-UPDRS scores without explicitly determining whether these changes exceed MCID thresholds. Therefore, some reported benefits may be statistically detectable but of uncertain relevance to daily mobility, fall risk, independence, or quality of life. To further contextualize the clinical relevance of representative exercise trials, key motor outcome changes and their MCID-based interpretation are summarized in Table 2.

**Table 2:** Summary of key quantitative biomarkers in PD exercise intervention research.

Biomarker category	Specific indicators	Core function/significance	Key evidence	Detection sample/ technology	Application value
Neurotrophic factors	BDNF	Promotes neuronal survival, differentiation, and synaptic plasticity; may reflect exercise-related neurotrophic responses, but its direct relationship with clinical improvement in PD remains uncertain.	1. Systematic review & meta-analysis (Kaagman et al., 2024): exercise (treadmill, rowing machine, etc.) significantly increased serum BDNF in PD patients, synchronized with enhancements in UPDRS scores, balance, and walking distance; 2. Meta-analysis (Rahmani et al., 2019): PD patients have lower serum BDNF than healthy controls; its downregulation correlates with motor symptom severity.	Serum, cerebrospinal fluid	1. Objectively quantify the intensity of neurotrophic support from exercise to the brain; 2. Provide candidate molecular evidence for exercise-related neuroplastic adaptation, while causal links with disease modification remain unproven.
Pro-inflammatory cytokines	TNF-α; IL-1β; IL-6	Key mediators of neuroinflammation; their levels tightly correlate with PD progression; assess exercise's anti-neuroinflammatory effects.	MPTP-driven PD mouse model: 1. Voluntary wheel running (Leem et al., 2023) inhibited microglial activation, decreased brain TNF-α, IL-1β expression; 2. Treadmill exercise (Wang et al., 2021) linked to TLR4/NF-κB anti-inflammatory pathway, explaining mechanism for behavioral enhancement.	Brain tissue (animals), blood	1. Precisely assess the anti-neuroinflammatory effects of exercise at the molecular pathology level; 2. Build the link between exercise-driven behavioral promotion and anti-inflammatory molecular pathways, identifying the mechanism of action.
Oxidative stress markers	8-iso-PGF2α; MDA	Indicates the degree of lipid peroxidation damage *in vivo*; assesses the. Notably regulatory effect of exercise on persistent oxidative damage in PD.	1. Acute exhaustive exercise can transiently increase markers (Nasca et al., 2010; triggering adaptive response); 2. Long-term low-intensity AE (Bouzid et al., 2014): decreased post-exercise blood MDA levels in the elderly, while enhancing SOD activity.	Urine (8-iso-PGF2α), blood (MDA)	1. Distinguish the acute (transient increase) vs. long-term (decreased baseline damage) effects of exercise on oxidative stress; 2. Assess whether exercise can mitigate persistent oxidative damage in PD because of factors, such as abnormal dopamine metabolism.
Imaging biomarkers	BOLD signal (fMRI); Cerebral blood flow perfusion (autoradiography/fMRI); Cerebral glucose metabolic rate (18F-FDG PET)	Non-invasively quantifies brain function, local blood flow, and neuronal energy metabolism status; visually reflects exercise-driven remodeling of brain networks.	1. Rat PD Model (Wang et al., 2013): exercise increased blood flow perfusion in dorsal striatum, motor cortex, suppressed abnormal hyperactivation in compensatory brain regions; 2. Human study (Matthews et al., 2018): FDG-PET-derived Parkinson's disease-related metabolic patterns may serve as candidate imaging biomarkers for clinical trials, but their role in monitoring exercise response remains insufficiently validated, reflecting modifications in neuronal energy supply.	Live brain (fMRI/ autoradiography/ PET)	1. Visually display exercise-driven enhancement in functional connectivity and energy supply in PD key brain regions (e.g., striatum and motor cortex); 2. Provide physiological evidence that may help explain exercise-related changes in motor or cognitive function.

BDNF, Brain-Derived Neurotrophic Factor; UPDRS, Unified Parkinson's Disease Rating Scale; TNF-α, tumor necrosis factor-alpha; IL-1β, interleukin-1 beta; IL-6, interleukin-6; MPTP, 1-methyl-4-phenyl-1,2,3,6-tetrahydropyridine; TLR4, Toll-like receptor 4; NF-κB, nuclear factor kappa B; 8-iso-PGF2α, 8-iso-prostaglandin F2α; MDA, malondialdehyde; AE, aerobic exercise; SOD, superoxide dismutase; BOLD signal, blood oxygenation level-dependent signal; fMRI, functional magnetic resonance imaging; 18F-FDG, 18F-fluorodeoxyglucose; PET, positron emission tomography.

Overall, available evidence suggests an intensity-dependent but non-linear pattern. Low-intensity exercise may be most suitable for improving tolerance, mobility, adherence, and safety in frail or advanced patients. Moderate-intensity exercise may provide the most practical balance between safety and functional benefit for many patients with mild-to-moderate PD. High-intensity exercise may provide additional benefits for cardiorespiratory fitness and selected motor outcomes in appropriate candidates, but its long-term efficacy and safety, as well as superiority over moderate-intensity training, remain uncertain. Current evidence is limited by inconsistent intensity definitions, heterogeneous patient populations, short intervention periods, and insufficient reporting of clinically meaningful outcomes. Table 3 shows the interpretation of motor outcome changes in representative Parkinson's Disease exercise trials using Minimum Clinically Important Difference Thresholds.

**Table 3:** Interpretation of motor outcome changes in representative Parkinson's disease exercise trials using minimum clinically important difference thresholds.

References	Population	Intervention	Duration	Motor outcome change	MCID interpretation
(Schenkman et al. 2018)	*De novo* PD	High-intensity treadmill training	6 months	UPDRS motor worsening was smaller in the high-intensity group than in usual care	The result suggests attenuation of motor worsening rather than symptomatic improvement; clinical relevance should be interpreted against UPDRS/MDS-UPDRS motor MCID thresholds
(Van Der Kolk et al. 2019)	Mild PD	Home-based aerobic exercise	6 months	Between-group difference in off-state MDS-UPDRS motor score favored aerobic exercise	The magnitude appears comparable to published MCID estimates for MDS-UPDRS Part III
(Chang et al. 2018)	Early-stage PD	Low-intensity cycling	8 weeks	UPDRS-III improved in both on- and off-medication states	Reported changes appear to exceed minimal clinically important thresholds, but interpretation is limited by the lack of a parallel control group
(Li et al. 2012)	Mild-to-moderate PD	Tai Chi	24 weeks	Balance and fall outcomes improved; UPDRS-III also improved	MCID for UPDRS-III is secondary; clinical relevance is mainly supported by postural stability and fall reduction

##### 3.2.1.2 Aerobic exercise vs. resistance training

Within PD exercise intervention approaches, AE and anaerobic exercise represent two mainstream rehabilitation strategies with distinct physiological processes and clinical effects. Evidence confirms that both can mitigate PD-associated motor symptoms to some extent, but their pathways of action, specific symptom domains promoted, and suitable populations differ significantly. Hence, carefully delineating the differences between these two categories of exercise interventions is critical for enhancing personalized PD rehabilitation plans.

AE, such as brisk walking, swimming, and cycling, can mainly improve cardiopulmonary capacity, promote cerebral blood flow and oxygenation, and thus facilitate neural function and motor recovery. For example, (Zhou et al. 2022), in a systematic review and network meta-analysis, pointed out that short-term high-intensity AE (e.g., 3 times/week, 30–45 min/session, and intensity 70–85% max HR) significantly improved UPDRS-III scores in PD patients, outperforming conventional training, notably in gait and coordination. A meta-analysis by (Zhen et al. 2022) further demonstrated the beneficial effects of AE on balance, walking endurance (e.g., 6-min walk test), and overall motor capacity. This confirms that AE has multifaceted beneficial effects on enhancing motor capacity in PD patients. Moreover, a randomized controlled trial (RCT) by (Johansson et al. 2022) suggested that long-term AE can increase functional connectivity between the anterior frontal–striatal circuits and sensorimotor cortex, implying a role for neuroplasticity mechanisms. Nevertheless, it remains unclear whether AE's effects on postural stability and anti-gravity muscles in advanced PD patients are significant, and whether these effects are regulated by disease duration, severity, or individual cardiopulmonary fitness. Hence, future research directions should focus on the effects of AE across different PD stages and longitudinal evidence for its neurophysiological mechanisms.

Conversely, anaerobic exercise, such as RT, can mainly improve muscle strength, endurance, and postural control, as well as decrease muscle rigidity and reduce fall risk. For instance, a systematic review by (Dibble et al. 2009), pointed out that anaerobic interventions can markedly promote balance and postural stability in PD patients, with effects more significant in mid- to late-stage patients. A study by (Ustinova et al. 2011) also pointed out that RT may increase gait speed, stride length, and movement initiation speed.

Overall, AE and anaerobic exercise have different emphases in PD rehabilitation: AE is suitable for early-stage patients, centering on overall motor capacity and neuroplasticity promotion, whereas anaerobic exercise is more applicable to mid- to late-stage patients, aiming to increase muscle strength, improve postural control, and decrease fall risk. Most current evidence is restricted to short-term effects and analysis of single exercise types. Future studies should delve into multimodal interventions combining AE and RT, centering on long-term effectiveness and personalized adaptation approaches.

##### 3.2.1.3 Exercise duration and frequency

In non-pharmacological intervention approaches for PD, exercise therapy has received widespread attention because of its advantages for motor symptoms. Significant evidence has confirmed that the duration and frequency of exercise interventions are key factors impacting effectiveness. Long-term, regular exercise training shows greater and more sustained benefits than short-term interventions for muscle strength, gait, balance, as well as for neuroplasticity and functional reorganization of brain networks (Mak et al., 2017; Chortane et al., 2024).

Notably, long-term exercise interventions lasting 12 weeks or more can more effectively increase muscular endurance, promote motor coordination, and drive neural functional adaptations. Particularly, a systematic review by (Mak et al. 2017) pointed out that integrated AE and RT programs, lasting for over 12 weeks, significantly increased balance, walking speed, and limb muscle strength in PD patients. This reveals that long-term exercise can not only help with symptom control but may also mitigate disease progression, possibly through processes involving increased brain network plasticity. Nevertheless, it remains unclear whether responses to the same exercise regimen differ among PD subtypes and how specific molecular pathways of neuroplasticity are regulated by exercise parameters. Hence, future studies should focus on the neurobiological mechanisms of exercise intervention and their application potential in personalized therapy. Conversely, short-term exercise interventions can mitigate motor symptoms to some extent, but the effects are generally transient and unlikely to induce sustained neural functional enhancements. For instance, (Chortane et al. 2024) compared long-term vs. short-term training effects, finding significant improvements in balance and quality of life only in the long-term group. This further encourages the notion that exercise interventions need sufficient duration to achieve substantive neuroplastic adaptation.

In addition to duration, exercise frequency is another critical variable impacting effectiveness. Literature confirms that moderate- to high-intensity exercise performed 3–5 times per week more effectively promotes gait, balance, and muscle strength, and slows disease progression. (Cordani and Mosconi 2024), in a Cochrane review, indicated that regular exercise frequency is tightly linked to improved quality of life and sustained motor capacity in patients. This stresses the significance of the “dose” effect of exercise intervention in the management of PD. Nevertheless, the optimal exercise intensity and frequency may vary by stages of disease progression, exercise baseline, and comorbidities, and evidence on the frequency effects of different exercise modes (e.g., Tai Chi and AE, RT) is lacking. Hence, future studies should focus on refining the definition of exercise parameters and personalized adaptation approaches.

From a mechanistic perspective, long-term regular exercise may exert functional enhancements through numerous pathways, comprising enhancing cerebral blood flow, regulating neurotransmitters, and promoting synaptic plasticity. (Petzinger et al. 2013) stressed that AE can remodel neural circuits linked to motor and cognitive function, hence enhancing overall neurobehavioral performance. Moreover, an RCT by (Chang et al. 2024) also reported that both 12 weeks of Tai Chi and AE significantly improved neurocognitive performance in early PD patients, further supporting the brain-remodeling effects of long-term exercise.

Overall, current evidence encourages the demonstrated benefits of long-term (≥12 weeks), regular (3–5 times/week) exercise interventions for alleviating motor symptoms, improving neural function, and enhancing quality of life in PD patients. Future studies should investigate the basis for formulating personalized exercise prescriptions by identifying optimal exercise parameters and integrating neuroimaging and molecular markers. This will deepen our understanding and enhance the precise application of exercise therapy in the comprehensive management of PD.

#### 3.2.2 Comparison of exercise types

The comparison of exercise categories should focus on the intervention processes, differences in effects, and clinical evidence of various forms of exercise for PD patients. This will systematically explain the key value and applicable scenarios of different exercises in PD rehabilitation. A comparative analysis of the intervention effects of different exercise categories on PD patients is reported in Table 4.

**Table 4:** Comparative analysis of intervention effects of different exercise categories in Parkinson's disease (PD).

Exercise type	Core mechanism	Main effects	Key conclusions	References
Aerobic exercise (AE)	Improves cardiorespiratory fitness, systemic circulation, cerebral oxygen delivery, cerebral blood flow, and functional connectivity.	May improve global motor function, gait performance, aerobic capacity, and selected cognitive–motor outcomes. Effects on balance and fall prevention may be weaker when AE is used alone.	Moderate- to high-intensity AE may benefit individuals with early-to-moderate PD when tolerated. Combined AE and resistance training may provide broader benefits than AE alone. Short-term AE may affect functional connectivity, whereas longer interventions may be required for more stable clinical effects.	Griffith et al., 2025; Chen and Chen, 2025; Johansson et al., 2022; Wang et al., 2015; Borrione et al., 2014
Resistance training/strength training (RT/ST)	Increases lower-limb strength, rate of force development, joint stability, and neuromuscular control related to posture and gait.	May improve strength, rigidity, gait speed, stride length, postural control, and fear of falling.	RT/ST is useful for patients with weakness, postural instability, and fall risk. Effects may vary by disease stage, baseline strength, cognitive status, and training progression.	Sherrington et al., 2019; Garber et al., 2011; Schlenstedt et al., 2015; Silva-Batista et al., 2018; Hirsch et al., 2003
Balance training, Tai Chi, Pilates, and virtual reality balance training	Enhances postural control, sensorimotor integration, coordination, weight shifting, and task-specific balance strategies.	May improve balance, gait stability, dynamic postural control, and fall-related outcomes. Tai Chi has relatively strong evidence for balance and fall reduction.	Balance-oriented exercise is especially relevant for patients with postural instability or gait impairment. VR-based approaches may improve engagement, but long-term evidence remains limited.	Li et al., 2012; Tsang, 2013; Çoban et al., 2021; Lin et al., 2016
High-intensity interval training (HIIT)	Provides vigorous cardiovascular and muscular stimulus and may induce metabolic and neuroplastic adaptations.	May improve cardiorespiratory fitness, VO_2_peak, muscular endurance, and selected motor outcomes in suitable patients. Evidence for balance, falls, and advanced PD is limited.	HIIT can be feasible and safe under appropriate screening and monitoring, but should not be generalized to frail patients or those with high fall, cardiovascular, autonomic, or cognitive risk. Long-term comparative efficacy remains uncertain.	Schenkman et al., 2018; Kathia et al., 2024; Sena et al., 2023; Harvey et al., 2019

##### 3.2.2.1 AE

As a key non-pharmacological intervention, the mechanisms by which AE can promote motor capacity in PD patients mainly include increased cardiopulmonary endurance and systemic circulation. This facilitates brain tissue oxygenation and nutrient supply, creating favorable conditions for the maintenance and recovery of motor capacity (Chen and Chen, 2025). Building on this, current clinical evidence has further compared the specific effects of different exercise regimens on the prognosis of early PD patients. For instance, a study by (Chen and Chen 2025) reported that, in comparison to AE alone, integrated AE and RT had significant benefits in facilitating patients' motor capacity, postural stability, cognitive processing speed, and peak oxygen consumption, with notably significant effects on key motor symptoms such as tremor, rigidity, and bradykinesia. This implies that comprehensive exercise interventions may achieve multi-dimensional functional enhancement through synergistic multi-system actions.

In addition to exercise type, intensity is another key variable impacting intervention findings. (Griffith et al. 2025) compared the effects of 6 months of high-intensity (80–85% max HR) vs. moderate-intensity AE on early PD patients, demonstrating that high-intensity training not only significantly strengthened AC but also effectively ameliorated motor symptoms. Notably, high-intensity exercise was well-tolerated and clinically effective even in patients with cardiac autonomic dysfunction. This confirms the crucial clinical potential of high-intensity AE for early PD patients. From a neural perspective, AE may enhance neuroplasticity by regulating cerebral blood flow and functional connectivity. (Johansson et al. 2022) reported that a single session of AE increased functional connectivity in the prefrontal cortex of PD patients, and this modification correlated with improved motor execution ability. In animal experiments, (Wang et al. 2015) further uncovered that long-term AE can enhance resting-state functional connectivity in neural circuits involved in motor control (including the motor cortex, thalamus, and basal ganglia), implying that exercise may provide a structural basis for functional enhancement by driving neural circuit reorganization.

Although current evidence supports the multifaceted advantages of AE for PD patients, differences exist in the design and effects of various exercise protocols. For example, short-term exercise (e.g., 20-min brisk walking) can significantly increase prefrontal functional connectivity, while long-term intervention (e.g., ≥12 weeks) is more conducive to structural remodeling of neural circuits (Johansson et al., 2022). Conversely, resistance training (RT) alone can improve muscle strength but has restricted effects on key symptoms such as tremor and bradykinesia (Borrione et al., 2014). Nevertheless, it remains unclear whether PD patients at different disease stages display heterogeneous responses to exercise intensity and type, and whether the causal pathway between neuroplastic modifications and clinical symptom enhancement is regulated by individual baseline brain network features. Future research should focus on systematically elucidating the personalized association processes between exercise intervention response and brain plasticity modifications by integrating multimodal neuroimaging markers with long-term follow-up data, thereby providing a theoretical basis for developing precision rehabilitation approaches.

Overall, AE, particularly moderate- to high-intensity types, may mitigate motor symptoms and enhance neural functional reorganization in PD patients. Future studies should systematically compare the sustainability of long-cycle vs. short-cycle exercise protocols and combine multimodal evaluation approaches, seeking to offer more targeted non-pharmacological exercise intervention recommendations for PD patients at different disease stages.

##### 3.2.2.2 RT and ST

ST is a crucial approach in PD rehabilitation interventions, with demonstrated effects on enhancing patients' muscle strength and endurance, facilitating postural control, and reducing fall risk. This confirms that ST not only helps mitigate muscle rigidity and increase joint range of motion but also can significantly increase motor capacity, particularly in lower limb control (Sherrington et al., 2019).

The main goal of ST is to enhance muscle strength, particularly in key lower limb muscle groups, which is critical for maintaining body balance and motor capacity. Relevant literature has shown that this type of training may mitigate muscle atrophy and may increase motor control ability (Sherrington et al., 2019). A meta-analysis by (Sherrington et al. 2019) further reported that ST can decrease fall incidence by 23% and fall risk by 15%, underscoring its significant role in fall prevention. Additionally, the American College of Sports Medicine recommends that adults perform ST for major muscle groups 2–3 times per week to maintain joint range of motion and overall functional level (Garber et al., 2011). This confirms that regular ST is a significant foundation for managing motor symptoms in PD patients. Nevertheless, it remains unclear whether patients with different PD subtypes or disease progression stages display differential responses to standardized ST protocols, which may be a main reason for the heterogeneity in intervention effects. Hence, future studies should focus on personalizing training parameters based on individual clinical features.

Concerning postural control, ST can directly improve balance by enhancing muscle strength and joint stability. (Schlenstedt et al. 2015), in an randomized controlled trial (RCT) comparing RT and balance training, uncovered that the RT group reported more significant enhancement on the Fullerton Advanced Balance (FAB) scale, and this enhancement was closely linked to enhanced rate of force development and improved gait variability. This implies that RT may be superior to balance training alone in facilitating postural control. Moreover, a study by (Silva-Batista et al. 2018) reported that instability-based RT significantly increased balance ability and decreased fear of falling. Nevertheless, it remains unclear whether the long-term maintenance effect of ST on postural stability in PD patients is regulated by cognitive decline or disease progression speed. Hence, future research should focus on the mechanisms by which multi-dimensional factors, such as cognitive–motor interactions, influence training effects.

ST also shows significant advantages in reducing fall risk. (Hirsch et al. 2003) compared the effects of high-intensity RT and balance training, demonstrating that both improved balance, but RT had greater benefits in muscle strength enhancement, and the effects persisted for up to 4 weeks after training cessation. This confirms that RT may decrease fall risk through dual pathways of muscle strength enhancement and neural adaptation. Although current evidence supports its effectiveness, effect sizes vary across studies; for instance, (Schlenstedt et al. 2015) stressed the independent benefit of RT, while other studies reveal that integrated training might be more beneficial (Schlenstedt et al., 2015; Silva-Batista et al., 2018). This confirms that training approaches should be flexibly tailored to specific rehabilitation goals. Nevertheless, it remains unclear how RT can achieve optimal synergy with emerging interventions, such as neuromodulation and pharmacological therapy. Hence, future studies should focus on comparative effectiveness studies of multimodal intervention protocols to develop optimal combination models.

Overall, ST, as a significant non-pharmacological approach for PD symptom control, can effectively improve muscle strength, postural control, and overall motor capacity, hence reducing fall risk and enhancing quality of life. Through systematic implementation, it offers a solid basis for PD rehabilitation, but further investigation is required for personalized protocol optimization and the maintenance of long-term effectiveness.

##### 3.2.2.3 Balance training

Balance training is a critical component of PD treatment, demonstrating significant effects in facilitating postural control, reducing gait instability, and reducing fall risk. Different categories of balance training, such as Tai Chi, pilates, and virtual reality (VR) balance games, have been demonstrated to significantly enhance balance and motor capacity in PD patients.

Tai Chi, a form of exercise integrating physical coordination and breath control, has been extensively utilized for balance training in PD patients. An RCT by (Li et al. 2012) uncovered that Tai Chi exercise significantly strengthened postural stability in PD patients, with notably specific effects on reducing tremor and bradykinesia. This study reported that PD patients engaging in Tai Chi performed better in balance tests (e.g., maximum excursion and directional control) than the control group receiving RT and stretching exercises. More importantly, Tai Chi exercise not only strengthened balance but also decreased fall incidence, and this effect was sustained for 3 months after training cessation (Li et al., 2012). (Tsang 2013) have further indicated the enhancement of balance ability by Tai Chi in PD patients, particularly concerning gait, dynamic step length, and stride. In comparison to RT and stretching, Tai Chi showed stronger benefits in enhancing postural stability and significantly decreased fall incidence over a 6-month intervention period. In addition to Tai Chi, Pilates is also an effective balance training strategy, advantageous for facilitating coordination, flexibility, and motor capacity. In PD patients, Pilates training offers significant benefits. Moreover, (Çoban et al. 2021) proved that clinical Pilates improved balance and postural control in patients with PD and may be a feasible balance-oriented intervention. This study emphasized the significance of Pilates in facilitating lower limb strength and enhancing postural control, particularly for motor capacity recovery in PD patients (Çoban et al., 2021). Besides, VR balance games, as an emerging training method, are also extensively utilized in balance training for PD patients. (Lin et al. 2016) proved that VR balance games significantly strengthened balance ability in PD patients, notably demonstrating significant enhancements in Berg Balance Scale (BBS) and Timed Up and Go Test (TUGT) scores. In comparison to traditional balance training, VR balance games, through their interactivity and engaging nature, can better enhance patient participation, hence facilitating training effectiveness.

Different forms of balance training demonstrate distinct therapeutic benefits for patients with PD. Tai Chi and Pilates primarily enhance postural stability and coordination, with significant benefits in managing gait instability, tremor, and bradykinesia (Li et al., 2012). Tai Chi, in particular, has been shown to effectively decrease fall risk, promote gait ability, and enhance motor capacity. Notably, Pilates emphasizes enhancing muscle strength and flexibility and has demonstrated beneficial effects on postural control and lower limb strength. VR-based balance game training represents an emerging training modality characterized by high interactivity and adjustability. VR training can be better tailored to individual patient needs while enhancing engagement and training results through real-time feedback (Lin et al., 2016). However, current evidence regarding VR-based balance games remains preliminary, so additional clinical studies are required to support their widespread clinical application.

Overall, balance training, such as Tai Chi and Pilates, has been shown to significantly promote balance ability, coordination, and motor capacity in PD patients. Tai Chi is particularly effective at improving gait and reducing fall risk, while Pilates excels at enhancing muscle strength and postural control. VR balance games, as an emerging training method, may enhance patient participation and training outcomes through interactivity and engagement. Future studies should continue to investigate the integrated effects of different categories of balance training to enhance treatment plans and promote the quality of life for PD patients.

##### 3.2.2.4 HIIT

HIIT, as a highly effective exercise intervention strategy, has demonstrated significant advantages in enhancing cardiovascular health, muscle strength, and overall physical performance. Recently, its application has extended to neurodegenerative diseases, demonstrating notable potential clinical value in alleviating motor symptoms in PD patients. This is evidenced by improved motor function, enhanced muscular endurance, and delayed disease progression.

(Schenkman et al. 2018) conducted an RCT on newly diagnosed PD patients, systematically assessing the influence of high-intensity interval treadmill training on PD motor symptoms. The study found that, compared to the usual care group, the HIIT group obtained significant enhancements in the Unified PD Rating Scale (UPDRS) motor scores, particularly in enhancing cardiopulmonary endurance and reducing bradykinesia. This confirms the clinical potential of HIIT as a feasible intervention for early-stage PD. Moreover, (Kathia et al. 2024) compared the effects of HIIT and moderate-intensity continuous training (MICT) on PD patients, demonstrating that HIIT had greater benefits in facilitating peak oxygen uptake (VO_2_peak) and knee extensor endurance. This finding reveals that HIIT may be superior to MICT in enhancing physical fitness and decreasing the decline of motor capacity in PD patients.

Nevertheless, it remains unclear whether HIIT has consistent benefits for PD patients across different disease stages and levels of disease severity, and whether its effects can be maintained during long-term interventions (Sena et al., 2023). Besides, although HIIT has shown promising improvements in multiple motor outcomes, its superiority over MICT in key functional parameters, such as balance and gait, remains unclear. Hence, future research should focus on elucidating dose–response relationships for HIIT and evaluating its synergistic effects and safety when integrated with pharmacotherapy. Concerning feasibility, Harvey et al.'s (2019) RCT demonstrated that although PD patients might experience tolerability issues with high-intensity training, HIIT remains highly executable and safe with appropriate monitoring and modifications. This confirms that HIIT can be a practical option for exercise rehabilitation in patients with PD, particularly when implemented under professional supervision.

Overall, HIIT shows significant potential in facilitating cardiorespiratory fitness, motor function, and muscular endurance in PD patients, with greater benefits over moderate-intensity training in enhancing AC and localized muscular endurance. However, its clinical implementation faces challenges including significant individual variability, inconsistent tolerance, and uncertain long-term efficacy. Future studies should focus on developing personalized intensity adjustment protocols, identifying the indications for HIIT across different stages of PD, and facilitating evidence-based assessment of its combination with multimodal treatment approaches.

#### 3.2.3 Physiological effects of exercise intensity

Exercise effectively improves motor capacity in PD patients through numerous physiological mechanisms, including regulating neuromuscular coordination, promoting neuroplasticity, and enhancing gait and motor control. Current evidence suggests that different exercise forms, such as AE, RT, and Tai Chi, may alleviate symptoms like bradykinesia, rigidity, and balance impairment, and enhance the recovery of mobility.

##### 3.2.3.1 Neuromuscular capacity regulation

Higher exercise intensity can improve neuromuscular coordination and motor control, thereby facilitating muscle strength, endurance, and motor responsiveness in PD patients, while alleviating symptoms such as bradykinesia and rigidity. This confirms that targeted approaches, such as ST and HIIT, show promise in enhancing neuromuscular capacity. ST, as a significant approach for facilitating neuromuscular capacity in PD patients, may enhance muscle strength and neuromuscular coordination. (Falvo et al. 2008) demonstrated that RT significantly decreased muscle rigidity in PD patients and increased lower limb strength, thereby facilitating postural control and balance, and reducing fall risk. Additionally, HIIT has also been shown to improve neuromuscular performance. For instance, (Kathia et al. 2024) reported that high-intensity interval cycling and moderate-intensity continuous cycling both improved selected outcomes in PD, with HIIT showing additional benefits for knee-extensor endurance.

##### 3.2.3.2 Increased neuroplasticity

Besides neuromuscular regulation, high-intensity exercise can also promote the release of neurotrophic factors (e.g., BDNF) and enhance neural connections between the cerebral cortex and basal ganglia, thereby promoting motor function recovery (Erickson et al., 2022). This suggests that exercise-related BDNF modulation may contribute to neuroplastic adaptation, although a direct causal relationship with clinical improvement remains unproven. Nevertheless, individual differences in BDNF response and its direct association with clinical symptom improvement have not been fully established. Future studies should focus on the quantitative correlation between BDNF dynamics and specific exercise parameters (e.g., intensity, type, and duration), as well as its predictive value in personalized rehabilitation protocols. AE is widely recognized to effectively enhance BDNF levels, thereby facilitating neuroplasticity and functional recovery. Gómez-Pinilla et al.'s (2002) review demonstrated that exercise increases BDNF expression, promoting synaptic plasticity and thus facilitating neural recovery. (Khalil 2025) further systematically reviewed and found that gait training and AE significantly increased BDNF levels and enhanced gait stability and coordination. Ashcroft et al.'s (2022) study also demonstrated that high-intensity AE significantly increased BDNF concentration, thereby promoting the reconstruction of motor and cognitive functions.

##### 3.2.3.3 Gait and motor coordination

Exercise effectively enhances motor coordination and activities of daily living in PD patients through improvements in postural control and gait stability. Comprehensive training combining AE with RT, as well as forms such as Tai Chi, have demonstrated significant effects in improving gait parameters (Song et al., 2025). This implies that multimodal exercise interventions may enhance gait control in PD patients through multi-pathway synergistic effects. Nevertheless, the specific mechanisms by which different exercise modalities target particular gait parameters (e.g., step frequency and stride length variability) remain to be fully elucidated. Notably, future studies should focus on the differential effects of various exercise categories on sub-gait indicators and develop personalized exercise prescriptions tailored to different stages of disease progression. (Martiš et al. 2025) reported that AE-RT combined training significantly enhanced postural stability and dual-task gait performance in PD patients, with notably significant effects in the off-medication state. Moreover, Lou et al.'s (Lou et al., 2025) meta-analysis indicated the effectiveness of Tai Chi in enhancing balance, lower limb mobility, and gait, supporting it as a low-risk, high-benefit exercise form notably suited for PD gait rehabilitation.

##### 3.2.3.4 Limitations of current evidence and future perspectives for motor symptoms

Although exercise interventions show potential for improving motor symptoms in PD, several limitations restrict clinical interpretation. First, exercise intensity is defined inconsistently across studies. Some trials use percentage of maximal heart rate or heart rate reserve (HRR), others use VO_2_peak, METs, workload, resistance load, or perceived exertion, and some studies use qualitative labels such as “moderate” or “high” without sufficient operational detail. This heterogeneity makes direct comparison between low-, moderate-, and high-intensity protocols difficult. Second, most studies include patients with mild-to-moderate PD, typically Hoehn and Yahr stages 1–3. Patients with advanced PD, severe postural instability, freezing of gait, cognitive impairment, orthostatic hypotension, or high fall risk remain underrepresented. Therefore, the safety and efficacy of moderate- to high-intensity exercise cannot be generalized to Hoehn and Yahr stages 4–5 without additional evidence. Third, many interventions are short term, commonly lasting 8–12 weeks, and long-term follow-up beyond 12 months remains limited. This makes it difficult to determine whether exercise produces durable symptom control, delays functional decline, reduces falls, or modifies disease trajectory. Fourth, many studies emphasize changes in motor scales but do not report MCID, fall rates, real-world physical activity, medication changes, adherence, or adverse events in sufficient detail. Future trials should standardize intensity reporting, include longer follow-up, stratify patients by disease stage and fall risk, and evaluate clinically meaningful outcomes rather than scale changes alone.

### 3.3 . Impact of exercise intensity on PD non-motor symptoms

#### 3.3.1 Quantification of non-motor symptoms

In addition to motor symptoms, PD patients generally experience a range of non-motor symptoms, such as depression, anxiety, cognitive dysfunction, and reduced sleep quality, which significantly impair overall quality of life. Therefore, accurate quantification of non-motor symptoms is essential for exploring the effects of exercise interventions.

Regarding mood disorders, depression and anxiety are common non-motor manifestations in PD and are usually assessed using scales such as the Hamilton Depression Rating Scale, Hamilton Anxiety Rating Scale, and Beck Depression Inventory. Evidence from older adults with major depression suggests that aerobic exercise can reduce depressive symptoms, sometimes with effects comparable to pharmacotherapy (Blumenthal et al., 1999). Other chronic-disease populations, such as dialysis patients, may also show mood benefits after low- to moderate-intensity exercise (Natale et al., 2019). However, these findings should be treated as indirect evidence when applied to PD. PD-related depression and anxiety may involve dopaminergic, serotonergic, noradrenergic, inflammatory, sleep-related, and disability-related mechanisms, and the dose–response relationship between exercise intensity and mood improvement in PD remains insufficiently established.

For cognitive function, the Montreal Cognitive Assessment and Mini-Mental State Examination are commonly used screening tools, with attention to executive function, working memory, and visuospatial performance. Some non-PD studies in older adults or MCI populations suggest that cognitively engaging exercise, Tai Chi, music-integrated movement, or task-oriented interventions may improve executive function and global cognition (Wang et al., 2022; Lee et al., 2022; Zhou, 2025). However, transferability to PD-related cognitive impairment is uncertain because PD cognition is strongly influenced by fronto-striatal dysfunction, medication status, disease duration, sleep disturbance, depression, and baseline cognitive reserve. Therefore, cognitive benefits reported in non-PD populations should be interpreted as hypothesis-generating rather than definitive evidence for PD.

Sleep quality is commonly assessed using the Parkinson's Disease Sleep Scale and Pittsburgh Sleep Quality Index. Sleep disturbance in PD is heterogeneous and may include insomnia, fragmented sleep, REM sleep behavior disorder, nocturnal akinesia, nocturia, restless legs symptoms, and excessive daytime sleepiness. Exercise may improve sleep quality through fatigue regulation, circadian entrainment, mood improvement, and autonomic modulation. (Chen and Chen 2025) reported that combined AE and RT was more effective than AE alone in improving sleep-related outcomes in PD. However, the specific effects of intensity, timing, frequency, and exercise modality on different PD sleep phenotypes remain unclear.

Overall, standardized tools such as HAM-D, MoCA, PDSS, and PSQI are useful for assessing non-motor symptoms, but current evidence should not be interpreted as showing that one exercise protocol can equally improve all non-motor domains. Depression, anxiety, cognition, sleep disturbance, fatigue, and autonomic dysfunction have distinct pathophysiological mechanisms and may require symptom-specific exercise strategies. Table 5 summarizes current assessment tools, intervention modalities, and limitations of the evidence.

**Table 5:** Summary of quantitative evaluation and exercise intervention literature for PD non-motor symptoms.

Non-motor symptom category	Assessment tools	Exercise intervention modality	Key literature findings	Pending issues
Mood disorders (depression, anxiety)	HAMD; HAM-A; BDI	AE; Low- to moderate-intensity exercise	1.16-week AE training significantly decreased HAMD and BDI scores in elderly depressed patients, with effects comparable to medication (Blumenthal et al., 1999); 2. Low- to moderate-intensity exercise enhanced depressive mood in dialysis patients, but its applicability in the PD population is unclear (Natale et al., 2019).	1. Neural processes and dose–response correlation of exercise intervention for PD comorbid depression are unclear; 2. Lack of literature on the specificity of exercise intervention effects for PD depressive symptoms and underlying biological pathways.
Cognitive dysfunction	MoCA; MMSE; Flanker Task (assesses executive function)	magic-based intervention; Tai Chi integrated with Music Therapy	1. MoCA's sensitivity in distinguishing early MCI from healthy controls may be inferior to MMSE (Wang et al., 2022); 2. 6-week magic-based intervention increased MoCA scores and executive function in elderly MCI group (Lee et al., 2022); 3. Tai Chi integrated with music therapy enhanced attention and executive control in elderly individuals with MCI (Zhou, 2025).	1. Transferability of cognitive enhancement effects to PD-associated cognitive dysfunction is unclear; 2. Differences in responses of different cognitive domains (memory, executive function) to exercise intervention are unknown; 3.The compatibility between PD cognitive dysfunction subtypes and exercise intervention programs, as well as the long-term maintenance effects, requires verification.
Decreased sleep quality	PDSS; PSQI	Combined AE + RT; AE alone	1. PD patients' PSQI scores were significantly higher than healthy controls and linked to disease severity (Hoehn & Yahr stage; Pezzini et al., 2024); 2. Combined AE + RT was more effective than AE alone in facilitating sleep quality in PD patients (Chen and Chen, 2025).	1. Specific effects of different exercise parameters (intensity, frequency, duration) on PD sleep stages (e.g., REM sleep behavior disorder) are unclear; 2. Need to investigate mechanisms of the exercise-sleep correlation (e.g., circadian rhythms, neurotransmitter regulation pathways).

HAMD, Hamilton Depression Rating Scale; HAM-A, Hamilton Anxiety Rating Scale; BDI, Beck Depression Inventory; AE, aerobic exercise; MoCA, Montreal Cognitive Assessment; MMSE, Mini-Mental State Examination; MCI, mild cognitive impairment; PDSS, Parkinson's Disease Sleep Scale; PSQI, Pittsburgh Sleep Quality Index; RT, resistance training; REM, rapid eye movement.

#### 3.3.2 Exercise intensity and mood regulation

Exercise intensity, a key parameter of exercise intervention, can significantly influence mood regulation by regulating intensity, duration, and frequency, and is notably applicable for mood control in PD patients. The mechanisms by which exercise intensity can impact mood regulation include, among others, neurotransmitter levels, neuroplasticity, and hypothalamic–pituitary–adrenal (HPA) axis function. Evidence from stress-related disorders suggests that exercise may regulate emotional and stress responses through interactions between oxidative stress and HPA-axis feedback; however, this evidence should be treated as indirect mechanistic support rather than direct clinical evidence in PD (Zhang and Kong, 2025).

Low-intensity exercise (e.g., walking or light AE) may reduce anxiety symptoms but has relatively limited effects on improving depressive symptoms. Its mechanism may involve reducing neuroinflammation and modulating neurotransmitter levels (e.g., serotonin and dopamine; Choudhary and Lee, 2018). Nevertheless, in PD patients, low-intensity exercise might modulate mood through similar pathways, but individual differences should be considered.

Moderate-intensity exercise (e.g., brisk walking and cycling) has broader effects on mood regulation. Previous studies suggest that moderate-intensity exercise can significantly enhance serotonin and dopamine levels, key neurotransmitters in mood regulation (Meeusen and De Meirleir, 1995). Moreover, moderate-intensity exercise can mitigate depressive symptoms by enhancing neuroplasticity, similar to the mechanism of antidepressant medication (Duman and Aghajanian, 2012). In PD patients, moderate-intensity exercise may alleviate mood symptoms by modulating neurotransmitter activity and improving synaptic function, with effects comparable to pharmacotherapy (Blumenthal et al., 2012). Systematic reviews further support the beneficial effects of exercise on depression, although the effect size may vary with study quality (Cooney et al., 2013). For anxiety symptoms, moderate-intensity exercise can reduce anxiety symptoms by enhancing cerebral blood flow and oxygenation, with effects comparable to psychotherapy (Herring et al., 2010; Hofmann and Smits, 2008).

High-intensity exercise (e.g., HIIT or running) can significantly affect mood regulation, notably in reducing depressive symptoms. Previous studies suggest that high-intensity exercise decreases the stress response of the HPA axis in a dose-dependent manner, reducing cortisol secretion and boosting its recovery, hence alleviating anxiety symptoms (Caplin et al., 2021). In older adults, high-intensity AE significantly decreased symptoms of depression, anxiety, and stress, with effects regulated by genotype (Gujral et al., 2024). Cohort studies further report that moderate-to-vigorous physical activity is linked to a lower incidence of depression, particularly in older adults with chronic diseases (Laird et al., 2023). A network meta-analysis pointed out that high-intensity exercise modalities (e.g., walking or yoga) are superior to other modalities in decreasing depressive symptoms, with treatment effects appearing to be intensity-dependent (Noetel et al., 2024). Additionally, exercise frequency can significantly affect outcomes, with moderate- to high-intensity training three times a week being effective in decreasing depressive symptoms (Chin et al., 2022).

Overall, exercise intensity, together with exercise duration and frequency, plays a critical role in mood regulation in PD patients. Low-intensity exercise mainly decreases anxiety, moderate-intensity exercise can comprehensively mitigate depression and anxiety, while high-intensity exercise performs prominently in depression control. Future studies should focus on developing personalized exercise interventions for PD patients to enhance mood regulation effects.

#### 3.3.3 Symptom-specific implications for exercise prescription

A single exercise protocol is unlikely to provide equivalent benefit across all non-motor symptoms in PD. For depression and anxiety, moderate-intensity aerobic exercise, group-based exercise, dance, Tai Chi, or aquatic exercise may be useful because these approaches combine physiological activation with social engagement and adherence support. For cognitive impairment, cognitively demanding exercise, dual-task gait training, dance, exergaming, or movement learning may be more relevant than simple repetitive exercise because they place greater demands on executive control and sensorimotor integration. For sleep disturbance, exercise timing should be considered, and vigorous exercise close to bedtime should generally be avoided. For fatigue, orthostatic symptoms, or prominent autonomic dysfunction, lower initial intensity, interval-based progression, seated or aquatic exercise, and closer monitoring may be safer. Therefore, non-motor symptom management requires matching exercise intensity and modality to the dominant symptom domain rather than prescribing intensity alone.

#### 3.3.4 Improvement of cognitive and brain function

Cognitive dysfunction is a common non-motor symptom in neurological disorders, such as PD, and exercise intensity, as a non-pharmacological intervention strategy, has attracted **considerable attention for its potential to improve cognitive function and brain health**. Significant evidence suggests that the effects of exercise vary depending on its intensity, duration, and type. This section systematically discusses its processes and empirical evidence across low, moderate, and high intensity exercise interventions, and reviews the current evidence and research limitations.

Low-intensity exercise has relatively modest effects on improving cognitive function. The underlying mechanisms may involve **improved cerebral blood flow** and reduced oxidative stress, providing support for basic cognitive health. Systematic reviews by (Singh et al. 2025) and (Tse et al. 2015) suggested that low-intensity exercise has beneficial effects on global cognition, memory, and executive function in **older adults**, **supporting its role as** an ideal choice for facilitating public health and maintaining basic cognitive function. This implies that for individuals with poor physical fitness, older age, or certain comorbidities, low-intensity exercise is a safe and feasible starting point, helping to build exercise habits and achieve initial health benefits. Nevertheless, it remains unclear whether the advantages of low-intensity exercise exhibit a “ceiling effect,” and whether its long-term effects are sufficient to delay or prevent the progression of neurodegenerative diseases.

Moderate-intensity exercise is currently considered the “optimal zone” for improving cognitive function, significantly enhancing working memory, executive function, and information processing speed. Evidence suggests that its processes include structural and functional changes in key brain regions (e.g., hippocampus and prefrontal cortex). For example, (Erickson et al. 2011) pointed out that AE can reverse age-associated hippocampal atrophy. More critically, moderate-intensity exercise effectively promotes the release of BDNF, a key molecule for enhancing neuroplasticity and forming new neural connections (Coelho et al., 2013). Ye M. et al.'s (2024) meta-analysis further suggested a dose–response correlation between exercise parameters (e.g., duration and frequency) and different cognitive domains, providing a basis for personalized exercise prescriptions. These findings suggest that moderate-intensity exercise acts through multiple synergistic mechanisms (structural, functional, molecular), **supporting its role as an important strategy for cognitive enhancement**. Nevertheless, as reported in the EXERT study (Baker et al., 2025), in patients with MCI, the cognitive enhancement effects of different intensity levels (moderate to high vs. low intensity) may not differ significantly. This implies that for groups with early cognitive impairment, adhering to regular exercise itself might be more critical than targeting a specific exercise intensity, and the underlying mechanism may need to be understood from **the perspective of** maintaining neural network stability rather than inducing massive changes.

High-intensity exercise shows strong potential in improving cognitive function, particularly executive function and information processing speed, in specific populations, such as PD patients. Its processes may go beyond the release of neurotrophic factors, focusing more on facilitating functional reorganization of brain networks, such as facilitating cerebral blood flow and enhancing functional connectivity in higher-order cognitive circuits, such as the frontal–basal ganglia pathways. Chang et al.'s (2025) meta-analysis reported that high-intensity exercise significantly strengthened executive function. This implies that for individuals who can tolerate it, high-intensity exercise may be an effective approach for substantial cognitive improvement. Nevertheless, a significant unresolved issue is the generalizability and safety of high-intensity exercise. Its advantages may be significantly regulated by factors such as age, disease severity, pharmacological treatment (e.g., levodopa dose), and baseline cognitive status (Chang et al., 2025). For example, Gobbi et al.'s (Gobbi et al., 2021) study found that overly complex high-intensity multimodal training was less effective than functional or cognitive training for PD patients. This strongly suggests that a “one-size-fits-all” high-intensity approach is not optimal, and future studies urgently require identifying which subpopulations benefit most and establishing rigorous safety guidelines.

Neuroimaging evidence offers a biological basis for the aforementioned behavioral findings. Functional magnetic resonance imaging (fMRI) studies have reported an exercise intensity-dependent correlation between exercise intensity and brain plasticity, with different intensity levels eliciting distinct changes in brain structural and functional connectivity. (Zhang et al. 2022) and (Mehren et al. 2019) provided evidence for this. Moderate- and high-intensity exercise have been shown to increase the synergistic activity of large-scale brain networks (e.g., default mode network and executive control network), which may be the neural basis for their enhancement of overall cognitive performance (especially executive function and memory), as summarized in the systematic review by (Moore et al. 2022). This confirms that exercise-driven cognitive enhancement is not vague, but is supported by observed neural circuit remodeling. Nevertheless, a current limitation is that most neuroimaging studies have small sample sizes and are either correlational or short-term intervention studies. It remains unclear how these functional connectivity changes specifically translate into increased cognitive function in daily life, and their long-term stability warrants further verification.

Overall, the effects of exercise intensity on cognition and brain function exhibit a clear intensity gradient and numerous mechanisms. Recent evidence supports integrating regular exercise, notably moderate-intensity exercise, as a key approach for maintaining and enhancing cognitive health. Nevertheless, this area still faces several key issues: First, personalizing optimal exercise prescriptions (intensity, type, frequency, duration) remains a major challenge, requiring consideration of individual age, health status, genotype, and cognitive baseline. Second, the molecular and neural circuit mechanisms of exercise benefits, particularly the precise pathways by which different intensities produce differential effects, are not completely elucidated. Finally, effectively translating laboratory findings into community and clinical settings as scalable, sustainable intervention protocols is key to realizing their maximum public health value. Future studies should aim to conduct large-scale, long-term, multimodal RCTs, integrated with advanced neuroimaging techniques and personalized data analysis, to ultimately achieve the goal of “precision exercise” for facilitating brain health.

#### 3.3.5 Limitations of current evidence and future perspectives for non-motor symptoms

The evidence for exercise effects on non-motor symptoms is less mature than that for motor outcomes. First, non-motor symptoms are biologically heterogeneous. Mood symptoms, cognitive impairment, sleep disturbance, fatigue, pain, and autonomic dysfunction are unlikely to respond to the same exercise protocol through a single mechanism. Second, many studies use different scales, intervention durations, and patient populations, limiting comparability across trials. Third, some evidence is extrapolated from non-PD populations, such as healthy older adults, patients with MCI, or patients with other chronic diseases. Such studies are useful for mechanistic hypotheses but should not be treated as direct clinical evidence in PD. Fourth, non-specific effects such as social interaction, therapist attention, expectancy effects, and improved general health may contribute to improvements in depression, sleep, and quality of life. These factors are difficult to separate from the specific physiological effects of exercise intensity. Fifth, long-term follow-up beyond 12 months is scarce, and advanced PD patients are underrepresented. Future studies should use symptom-specific inclusion criteria, standardized non-motor scales, adequate control conditions, longer follow-up, and stratification by medication status, cognitive status, disease stage, and autonomic symptoms.

### 3.4 Physiological and molecular mechanisms of exercise

#### 3.4.1 Exercise and neuroplasticity

**Figure 1:**
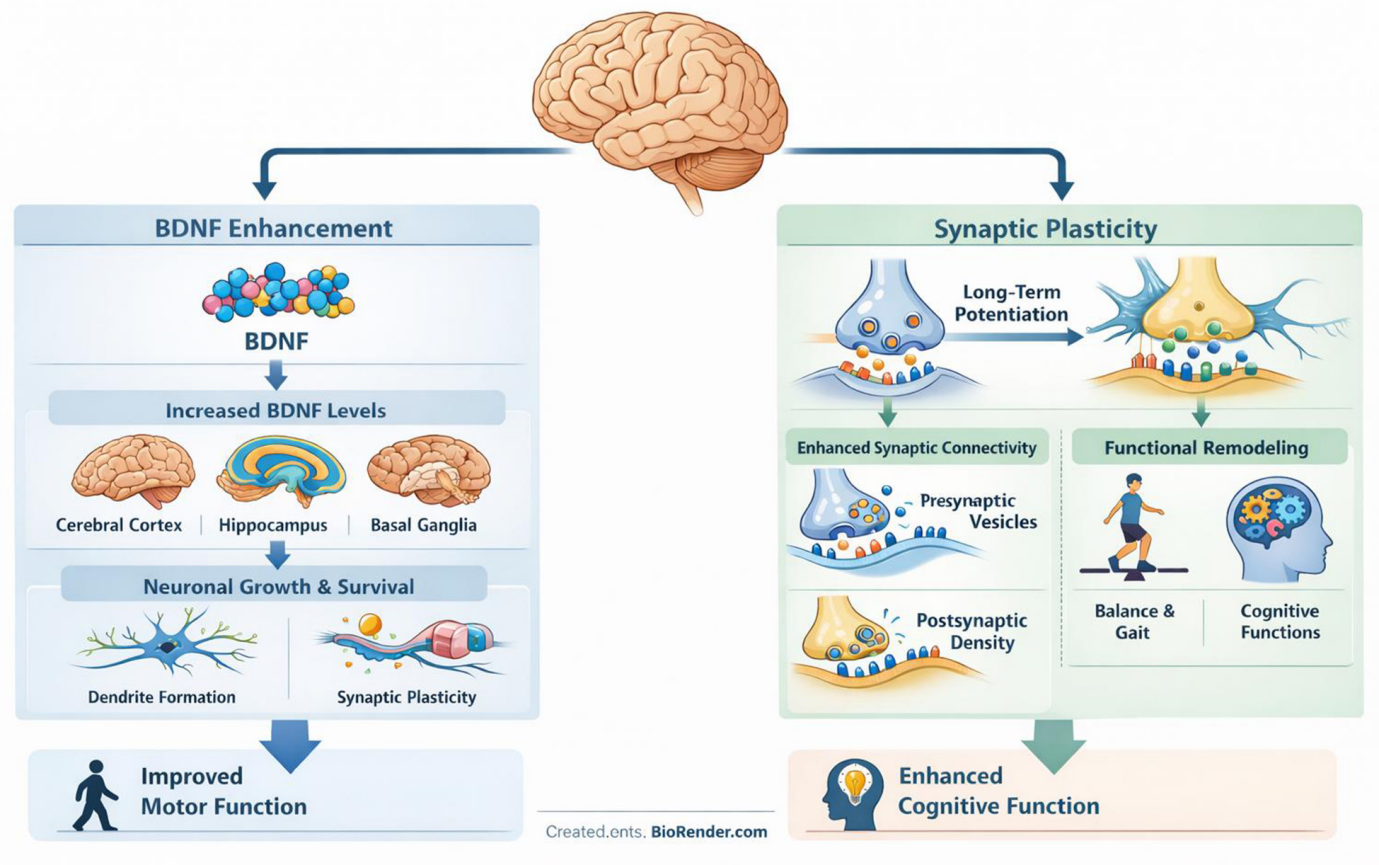
Mechanisms of exercise-driven neuroplasticity in Parkinson's disease.

Neuroplasticity refers to the capacity of the nervous system to adapt through changes in synaptic strength, functional connectivity, neuronal excitability, and network organization. Exercise may promote neuroplasticity in PD through neurotrophic signaling, motor learning, task-specific practice, and changes in cerebral perfusion. However, the causal pathway linking exercise intensity, biomarker changes, brain network remodeling, and clinical improvement remains incompletely established in humans. The putative mechanisms by which exercise may influence neuroplasticity in PD are illustrated in Figure 1.

This figure illustrates how exercise intensity may modulate BDNF signaling and synaptic plasticity, which may contribute to improvements in motor and cognitive outcomes in Parkinson's disease patients. It shows the increase of BDNF in the cerebral cortex, hippocampus, and basal ganglia, promoting neuronal growth and synaptic plasticity, which in turn enhances motor function. In the synaptic plasticity section, exercise strengthens synaptic connectivity and long-term potentiation, further improving cognitive functions and motor abilities.

##### 3.4.1.1 BDNF upregulation

BDNF is a key neurotrophic factor involved in neuronal survival, synaptic plasticity, and activity-dependent neural adaptation. Exercise may influence BDNF signaling, but the strength of evidence differs across experimental levels. Preclinical studies provide mechanistic support for exercise-induced changes in BDNF-related pathways, whereas human studies in PD mainly rely on peripheral BDNF measurements and clinical correlations. A small PD study reported an association between serum BDNF and cognitive function, but this relationship varied according to physical capacity and should be interpreted cautiously because of limited sample size (Khalil et al., 2017). A broader meta-analysis across neurodegenerative diseases also suggested that exercise can increase circulating BDNF, but heterogeneity across populations and protocols limits direct inference for PD (Ruiz-González et al., 2021).

More recent PD-specific evidence suggests that exercise may increase BDNF levels, and higher exercise intensity may be associated with greater BDNF change in some analyses (Wang et al., 2021; Paterno et al., 2024). However, BDNF responses vary across studies, and it remains unclear whether peripheral BDNF changes directly mediate improvements in motor or cognitive outcomes. Acute exercise studies also suggest that mature BDNF may increase after a single light-to-moderate treadmill session, whereas proBDNF responses may differ (Azevedo et al., 2022). Therefore, BDNF should be described as a promising biomarker and plausible mechanistic pathway rather than definitive evidence of exercise-induced neuroplastic adaptation in PD.

Overall, exercise-related modulation of BDNF may contribute to neuroplastic adaptation in PD, but the evidence remains heterogeneous. Future studies should combine standardized exercise intensity reporting, repeated BDNF measurements, clinical MCID-based outcomes, neuroimaging markers, and stratification by genotype, disease stage, medication status, depression, fatigue, and baseline physical capacity.

##### 3.4.1.2 Neuronal synaptic plasticity

Exercise intensity, as a key physiological stimulus, may enhance communication efficiency within neural circuits by facilitating synaptic connections between neurons. Literature confirms that long-term exercise intervention can significantly enhance synaptic plasticity, manifesting as structural remodeling (e.g., enhanced dendritic spine density) and functional enhancement (e.g., long-term potentiation), thereby facilitating neuronal responsiveness to external stimuli and information-processing precision (Franzén et al., 2019). Through this mechanism, exercise intensity has been indicated to significantly enhance motor capacity (e.g., balance and gait control) and cognitive function (e.g., executive function and spatial cognition) in PD patients, with the most pronounced effects in the early and middle stages of the disease (Abraham et al., 2018).

Recent clinical trials further support this view. For example, the HiBalance program developed by (Franzén et al. 2019) is a high-intensity, cognitively demanding balance training protocol that induces neuroplastic changes through progressive dual-task training. This study employed functional magnetic resonance imaging (fMRI) and BDNF as neuroplasticity indicators, pointing out that exercise intervention not only strengthens behavioral performance but may also modulate brain structure and function. Similarly, dynamic neurocognitive imagery (DNI) training, proposed by (Abraham et al. 2018), enhances kinesthetic imagery and anatomical proprioception, thereby significantly improving imagery ability, reducing disease severity, and boosting motor–cognitive function in patients with PD. These findings suggest that imagery training may serve as a complementary strategy to exercise-based interventions, synergistically promoting synaptic plasticity.

This suggests that multimodal intervention approaches that combine exercise intensity and cognitive training may mitigate multi-dimensional symptoms of PD via synaptic plasticity mechanisms. Nevertheless, it remains unclear how different exercise intensity parameters (e.g., intensity, frequency, and duration) specifically regulate the molecular pathways of synaptic plasticity, particularly the spatiotemporal dynamics of the BDNF–TrkB signaling pathway in exercise-driven synaptic remodeling (Franzén et al., 2019). Moreover, current evidence is largely limited to behavioral and imaging correlations, with limited evidence at the synaptic ultrastructural level (e.g., presynaptic vesicle cycling and postsynaptic density protein expression; Abraham et al., 2018). Hence, future research should adopt integrated approaches that combine quantitative modeling of exercise intensity with multi-scale neurobiological markers (e.g., molecular imaging and electrophysiological recordings) to reveal the causal mechanisms of synaptic plasticity in PD rehabilitation.

#### 3.4.2 Neuroinflammation and oxidative stress

**Figure 2:**
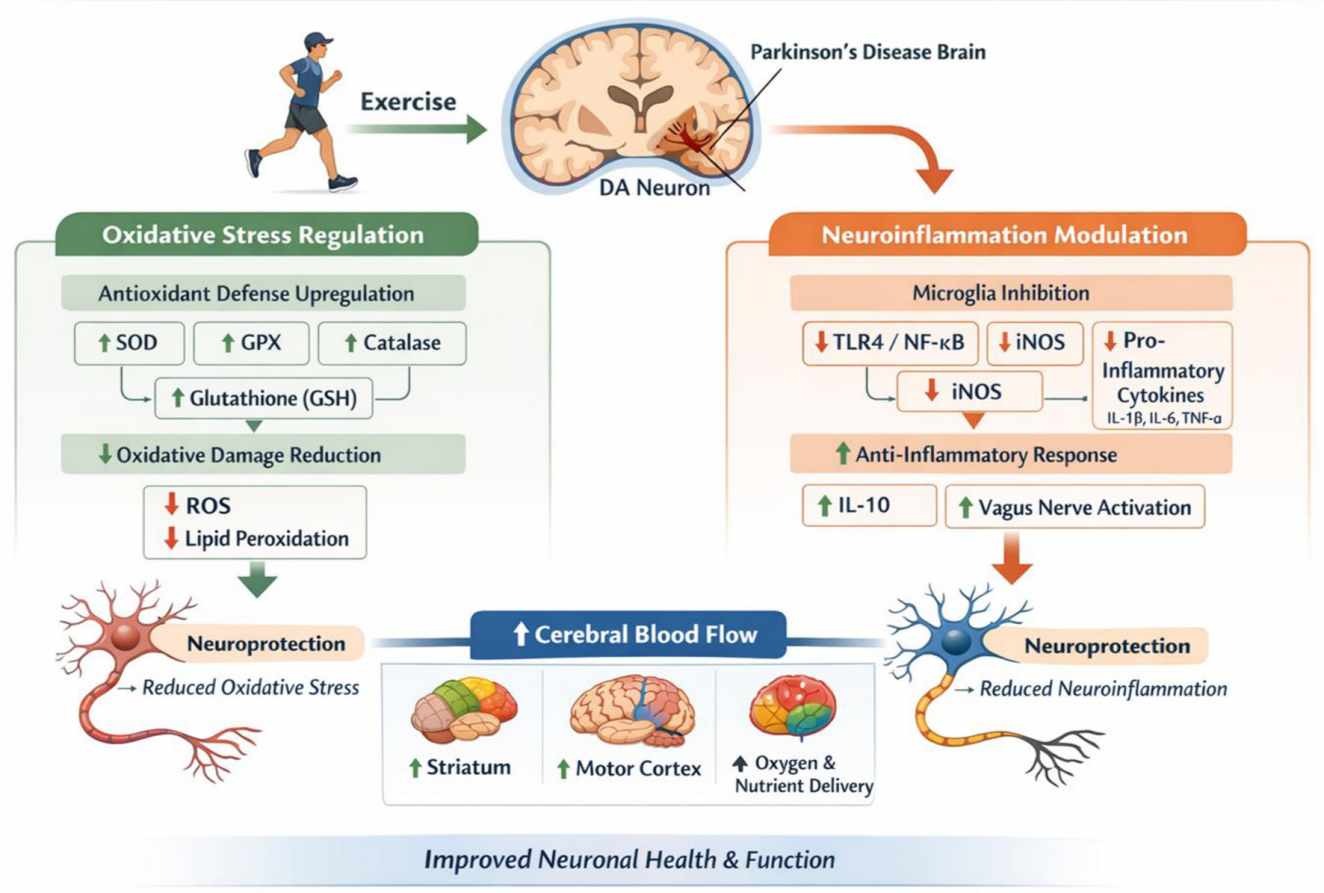
Mechanisms of exercise-driven improvement in neuroinflammation and oxidative stress in Parkinson's disease. Exercise exerts neuroprotective effects in Parkinson's disease (PD) by coordinately attenuating oxidative stress, suppressing neuroinflammation, and improving cerebral blood flow. On the oxidative stress side, regular exercise enhances endogenous antioxidant defenses by increasing the activity or expression of key antioxidant components, including superoxide dismutase (SOD), glutathione peroxidase (GPX), catalase (CAT), and glutathione (GSH), thereby reducing reactive oxygen species (ROS) accumulation and lipid peroxidation. On the inflammatory side, exercise suppresses microglial overactivation and inhibits the TLR4/NF-κB signaling pathway, leading to reduced expression of inducible nitric oxide synthase (iNOS) and lower production of pro-inflammatory cytokines, including IL-1β, IL-6, and TNF-α. Exercise may also promote a systemic anti-inflammatory milieu by enhancing vagal activity and increasing anti-inflammatory mediators such as IL-10. In parallel, exercise improves perfusion in PD-relevant motor regions, particularly the striatum and motor cortex, thereby facilitating oxygen and nutrient delivery and supporting neurovascular coupling. Together, these adaptations help preserve dopaminergic neuronal integrity and improve overall neuronal health and function in PD.

In the pathophysiology of PD, neuroinflammation and oxidative stress are two key driving factors, jointly exacerbating the damage and death of dopaminergic neurons. They are also key targets for the neuroprotective effects of exercise intervention. Evidence from gasotransmitter research, particularly hydrogen sulfide-related studies in PD, further supports the relevance of oxidative stress and inflammatory signaling in PD neuroprotection; however, such evidence should be interpreted as mechanistic context rather than direct proof of exercise efficacy (Ye Y. et al., 2024). The mechanisms by which exercise enhances neuroinflammation and oxidative stress in PD are illustrated in Figure 2.

##### 3.4.2.1 Regulation of oxidative stress

The pathophysiology of PD is closely linked to a persistent state of oxidative stress in the brain. Because mitochondrial dysfunction is a major upstream source of oxidative stress in PD, exercise-related modulation of mitochondrial biogenesis, mitophagy, respiratory chain function, and mitochondrial quality control may represent an additional pathway linking exercise intensity to neuroprotection (Kong et al., 2024b). Oxidative stress has also been discussed as a cross-system mechanism linking brain dysfunction with peripheral organ pathology, and oxidative biomarkers such as MDA, oxidized lipids, nitrotyrosine, and 8-OHdG may help characterize oxidative damage in neurodegenerative conditions including PD (Kong et al., 2024a). This state arises from an imbalance between the overproduction of reactive oxygen species (ROS) and the capacity of the body's antioxidant defense systems, ultimately causing damage and death of dopaminergic neurons. Hence, any intervention approach that may restore this balance holds profound significance for decreasing disease progression. Molecular hydrogen inhalation has similarly been proposed as a redox-modulating intervention in PD, but the available evidence remains preliminary and should not be extrapolated to exercise prescription without direct comparative trials (Ichikawa et al., 2024). Current evidence strongly confirms that regular exercise training, notably AE, is precisely such a powerful non-pharmacological strategy, with one of its key processes being the multi-pathway regulation of the body's oxidative stress levels.

The advantages of exercise are first reflected in its ability to systematically enhance the body's endogenous antioxidant defense network. Numerous studies in both humans and animals directly show that long-term aerobic exercise can upregulate the activity and expression of key antioxidant enzymes. For example, in healthy populations, studies have shown that 12 weeks of high-intensity endurance training significantly increased the activity of Superoxide Dismutase (SOD) and Glutathione Peroxidase (GPX) in erythrocytes. SOD is the first line of defense against oxidative damage, responsible for converting harmful superoxide anions into hydrogen peroxide, which is then further broken down into harmless water and oxygen by GPX and Catalase (CAT; Miyazaki et al., 2001). Similarly, animal experiments showed that aerobic exercise training also comprehensively increased the activity of different antioxidant enzymes, including CAT, GPX, and Mn-SOD, in the soleus muscle of young rats (Lambertucci et al., 2007). This means that individuals who have undergone exercise training have cells (including neurons) equipped with a more powerful “scavenger” system, capable of more efficiently neutralizing excess free radicals produced during exercise itself or under pathological conditions. For PD patients, this exercise-driven enhancement of antioxidant capacity has direct neuroprotective value. Studies directly targeting PD patients have shown that after 12 weeks of aerobic exercise intervention, blood CAT activity and Glutathione (GSH) levels were significantly increased in patients (Tsai et al., 2025). GSH is one of the most critical antioxidants in the brain, and its increased level is essential for maintaining the redox balance within neurons. The study also observed that the decrease in scores on cognitive function scales was attenuated in the aerobic exercise group, and the enhancement in antioxidant indicators was correlated with the maintenance of cognitive function, providing preliminary clinical support for an association between exercise-related antioxidant changes and cognitive outcomes.

Exercise not only “reinforces” defense capabilities but also “reduces the burden” at the source, i.e., decreases the burden of oxidative stress. High-intensity exercise itself is an acute oxidative stressor, but long-term training can induce an adaptive response in the body, thereby weakening the damage caused by subsequent oxidative stressors. One study reported this “exercise preconditioning” effect: After training, the increase in superoxide anion production by neutrophils during exhaustive exercise was reduced, and lipid peroxidation damage to erythrocyte membranes was also significantly decreased (Miyazaki et al., 2001). Lipid peroxides are key markers of oxidative damage. Another study also reported that after HIIT, the increase in Malondialdehyde (MDA) levels (a lipid peroxidation product) in young, active adults was greater than after MICT (Soylu et al., 2023). This suggests that exercise training can decrease cellular sensitivity to oxidative stress and decrease damage to biomacromolecules, such as lipids. In the context of PD, this means that exercise may help vulnerable neurons better withstand oxidative attacks caused by intrinsic pathology and extrinsic environmental factors. Notably, the antioxidant effects of exercise may be influenced by exercise mode, intensity, and individual differences. For example, studies have determined that HIIT may confer greater benefits in enhancing aerobic fitness and reducing oxidative stress markers (Soylu et al., 2023). Furthermore, body composition may also play a role, as those with a higher body fat percentage might be more prone to disturbances in antioxidant enzyme activity after strenuous exercise, underscoring the significance of personalized exercise plans for PD patients (Wiecek et al., 2025).

Overall, exercise may influence redox balance via enhancement of endogenous antioxidant capacity and reduction of oxidative damage. This molecular-level regulation may be one potential pathway through which exercise supports neuronal resilience and protects neuronal function, offering a solid scientific basis for its role as an effective adjuvant and complementary therapy to standard PD treatment, although evidence for disease modification in human PD is still limited.

##### 3.4.2.2 Regulation of neuroinflammation

Besides oxidative stress, neuroinflammation is another key factor driving the pathogenesis of PD. In the brains of PD patients, microglia (the brain's immune cells) are persistently activated, releasing large amounts of pro-inflammatory cytokines, such as tumor necrosis factor-alpha (TNF-α), interleukin-1 beta (IL-1β), and interleukin-6 (IL-6). These inflammatory mediators exacerbate the damage and loss of dopaminergic neurons (Alam et al., 2016). Current evidence suggests that regular exercise may attenuate inflammatory signaling, providing protection for the brain by suppressing neuroinflammation. However, evidence in human PD remains limited.

The most direct anti-inflammatory mechanism of exercise is to inhibit the overactivation of microglia and downregulate key pro-inflammatory signaling pathways. Studies conducted in a PD mouse model offer strong evidence for this. One study reported that 14 days of treadmill running not only significantly strengthened motor function in PD mice but also suppressed microglial activation (Wang et al., 2021). At the molecular level, exercise significantly decreased the expression of inducible Nitric Oxide Synthase (iNOS) and IL-1β in microglia and downregulated the key pro-inflammatory signaling pathway Toll-like receptor 4 (TLR4)/nuclear factor kappa B (NF-κB), ultimately leading to reduced levels of pro-inflammatory cytokines, such as IL-1β, IL-6, and TNF-α (Wang et al., 2021). This suggests that exercise may downregulate inflammatory signaling pathways implicated in PD models, creating a microenvironment unfavorable for neuronal damage. Clinical studies in PD patients have also observed similar anti-inflammatory trends. An RCT in PD patients uncovered that after 12 weeks of Qigong (a mind–body exercise) intervention, there was a trend toward decreased concentrations of IL-1β and IL-6 (Moon et al., 2024). The study also uncovered an association between modifications in inflammatory markers and enhancements in sleep quality, implying that exercise may mitigate non-motor symptoms in PD by reducing neuroinflammation, hence comprehensively enhancing patients' quality of life (Moon et al., 2024). Moreover, the anti-inflammatory effects of exercise may also be obtained by regulating the neuro-immune-endocrine network, a more systemic, indirect mechanism. A review concluded that regular physical exercise is key to maintaining a low-inflammatory state in the body (Cardinali, 2021). Additionally, a review on the impact of ω-3 fatty acids further elucidated a possible mechanism: Exercise can enhance parasympathetic nerve tone (vagus nerve activity). When the vagus nerve is activated, it releases the neurotransmitter acetylcholine, which can significantly inhibit the release of pro-inflammatory factors, such as TNF-α, IL-1β, and IL-6 (Das, 2000). Simultaneously, exercise can promote the production of anti-inflammatory cytokines (e.g., IL-10). This implies that exercise not only exerts direct effects on the brain but also indirectly creates a systemic anti-inflammatory environment that benefits the brain by regulating the autonomic nervous and immune systems (Das, 2000).

In preclinical PD models, exercise has been shown to downregulate inflammatory signaling pathways, including TLR4/NF-κB-related pathways. However, whether these mechanisms directly mediate clinical improvements in human PD remains uncertain.

##### 3.4.2.3 Enhancement of cerebral blood flow

PD is not only characterized by degeneration of the dopaminergic system but is also accompanied by extensive alterations in brain function and structure, among which abnormal cerebral blood flow perfusion is a significant feature (Xie et al., 2023). In PD, regions closely linked to motor control, such as the basal ganglia (especially the dorsolateral striatum) and motor cortex, generally display hypoactivity and hypoperfusion (Van Laere et al., 2004). Exercise has been reported to effectively enhance cerebral blood flow, promote the delivery of oxygen and nutrients, support compromised brain tissue, and facilitate its functional reorganization. Assessment of cerebrovascular reactivity, including CO_2_ vasomotor reactivity, may help interpret exercise-related perfusion changes, although it is not yet an established tool for exercise prescription in PD (Sensier et al., 2024).

Exercise-induced enhancement of cerebral blood flow is not a global, uniform increase, but rather a precise, functionally specific reorganization matching the demands of neural circuits. A study in a rat PD model suggested this: Utilizing three-dimensional cerebral blood flow imaging, the study uncovered that untrained PD model rats exhibited significant hypoactivation in the dorsal striatum and motor cortex during walking (Wang et al., 2013). Nevertheless, after 4 weeks of forced wheel running, blood flow perfusion in these key motor areas significantly increased. Concurrently, exercise also remodeled the activity of the entire motor network, suppressing the abnormally high activation in other brain regions (e.g., globus pallidus and subthalamic nucleus) resulting from compensatory mechanisms, and led to functional reorganization of the cerebellar–thalamic–cortical circuit (Wang et al., 2013). These findings suggest that exercise may partially normalize abnormal activity patterns in PD-related motor circuits, redirecting blood flow and neural activity back to the key areas needed for executing motor control, although most preliminary evidence remains preclinical. This enhancement in cerebral blood flow deeply synergizes with neuroplasticity. A review proposed a key mechanistic hypothesis: “neurovascular coupling.” When exercise can enhance neuroplasticity (e.g., regulating dopamine and glutamate transmission, changing synaptogenesis), the metabolic demands of neurons increase. To meet this increased energy demand, the brain responds by enhancing local cerebral blood flow (Petzinger et al., 2015). This coupling of “increased metabolic demand” and “increased blood supply” creates the needed conditions for the repair and functional enhancement of neural circuits. Hence, exercise may, through this synergistic mechanism, target specific neural circuits to induce advantageous remodeling.

#### 3.4.3 Quantitative biomarkers

In PD exercise intervention research, objective biomarkers may help characterize biological responses to training, but they are not yet validated for routine clinical prescription. Current candidate biomarkers can be grouped into several domains: neurotrophic markers, inflammatory markers, oxidative stress markers, neuroendocrine markers, PD pathology-related markers, and neuroimaging markers.

BDNF is one of the most frequently studied neurotrophic markers due to its role in synaptic plasticity, neuronal survival, and activity-dependent adaptation. Systematic reviews suggest that exercise may increase circulating BDNF in PD, but the magnitude of change varies substantially across protocols, and the relationship between peripheral BDNF and clinically meaningful symptom improvement remains uncertain (Wang et al., 2021; Paterno et al., 2024). Therefore, BDNF is promising for research but should not yet be considered a validated marker for clinical decision-making. Inflammatory markers, including TNF-α, IL-1β, IL-6, and CRP, are relevant because neuroinflammation is implicated in PD progression. Animal studies provide stronger mechanistic evidence than human trials, showing that exercise can suppress microglial activation and inflammatory signaling pathways such as TLR4/NF-κB (Salvatore et al., 2022; Leem et al., 2023). In humans with PD, inflammatory responses to exercise remain less consistent and may be influenced by age, comorbidities, medication, sleep, body composition, and baseline inflammatory status. Oxidative stress markers, including MDA, 8-iso-PGF2α, antioxidant enzyme activity, and glutathione-related indices, may help distinguish acute exercise stress from longer-term adaptive responses. However, evidence in PD remains limited, and findings from healthy older adults or non-PD populations should be interpreted as indirect (Nasca et al., 2010; Bouzid et al., 2014). Neuroendocrine markers, such as cortisol and autonomic-related measures, may be useful for monitoring stress response, fatigue, sleep disturbance, and tolerance to higher-intensity exercise. PD pathology-related markers, especially α-synuclein species in cerebrospinal fluid, plasma, or extracellular vesicles, are also promising, but their relationship with exercise response remains preliminary (Luthra et al., 2025). Neuroimaging markers, including fMRI-based functional connectivity, cerebral blood flow, FDG-PET metabolism, dopamine transporter imaging, and structural MRI, may provide more direct information about exercise-related brain network adaptation. Nevertheless, these measures are expensive, technically demanding, and not yet standardized for clinical exercise prescription.

Overall, the most promising future biomarkers for PD exercise programs include BDNF and related neurotrophic markers, inflammatory markers such as IL-6, TNF-α, and CRP, oxidative stress markers such as MDA and 8-iso-PGF2α, neuroendocrine markers such as cortisol, PD pathology-related markers such as α-synuclein, and neuroimaging markers of functional connectivity, perfusion, metabolism, and dopaminergic integrity. At present, these biomarkers should be considered research tools rather than established clinical monitoring instruments. A summary of candidate quantitative biomarkers is shown in Table 2.

### 3.5 Personalized treatment approaches by exercise intensity

Personalized treatment is a critical approach in modern medicine, particularly for complex diseases such as PD. Developing personalized treatment plans based on a patient's specific condition, symptoms, and requirements can significantly improve therapeutic outcomes. Exercise intensity, as a significant component of PD treatment, benefits from personalized plan design, which can enhance effectiveness and avoid adverse reactions from over-intervention. A personalized treatment approach for PD motor and non-motor symptoms by exercise intensity is reported in Table 6.

**Table 6:** Personalized treatment approach for PD Motor and non-motor symptoms by exercise intensity.

Personalized treatment key dimension	Target symptom type	Specific exercise intensity treatment plan	Key parameters/implementation points	Clinical considerations	References
Based on patient baseline features					
Exercise capacity differences	Motor symptoms (muscle strength, balance, gait)	**Early PD**: moderate- to high-intensity AE (non-contact boxing, running, cycling); **Mid-late PD**: low-intensity, low-impact exercise (gentle stretching, Tai Chi, seated ST).	**Early**: heart rate increase ~35%, VO_2_peak enhancement; **Mid-late**: key focus on “no fall risk,” emphasis on balance and joint scope of motion.	Avoid over-protection in early stage; strictly control impact movements in mid-late stage; dynamically monitor post-exercise fatigue (especially mid-late stage).	Griffith et al., 2025; Salvatore et al., 2022; Duñabeitia et al., 2025; Kerr et al., 2016
Disease severity (H&Y stage)	Motor symptoms (overall motor capacity, fall risk)	**H&Y 1–2**: comprehensive training (AE + RT); **H&Y stages 3–4**: maintenance capacity training (low/mid-frequency treadmill, slackline balance training, golf).	**H&Y 1–2**: 12 weeks per course, progressively enhancing load; **H&Y stages 3–4**: goal is “preventing complications,” avoid high-frequency training.	H&Y stages 3–4 need training with companion, focus on fall prevention; avoid extreme fatigue from pursuing “capacity enhancement.”	Demonceau et al., 2017; Pelosin et al., 2017; Santos et al., 2017; Bliss and Church, 2021
Impact of non-motor symptoms	Non-motor symptoms (depression/anxiety, cognitive dysfunction, sleep disorders)	**Depression/anxiety**: light AE (walking in supportive environment, aquatic exercise, Tai Chi); **Cognitive/sleep disorders**: mind–body training (Tai Chi, yoga).	Intensity controlled within “pleasurable” range, RPE 11–13; single session ≤ 45 min, avoid training within 1 h before sleep.	Avoid forced high-intensity exercise in emotionally unstable patients; prioritize building exercise confidence; simplify movement instructions and use repetition for those with cognitive impairment	Van Der Heide et al., 2020; Nocera et al., 2013
Individual quality-of-life needs	Motor/non-motor symptoms (matched as needed)	**Improve motor capacity**: AE + unstable ST (e.g., BOSU ball half-squats); **Improve mood/sleep**: Tai Chi, yoga (focus on breathing & relaxation).	**Improve motor capacity**: single session ≥60 min (combined AE + RT); **Improve mood**: 16-week course, 3 times/week.	Set goals with the patient to increase long-term adherence; if requires conflict (e.g., “want to enhance motor function, but significant anxiety”), prioritize decreasing non-motor symptoms.	Kola and Subramanian, 2023; Nocera et al., 2013; Silva-Batista et al., 2016
Parameterized intervention details					
Exercise intensity	Motor/non-motor symptoms (common)	**Standard intensity**: AE (60–80% HRmax/60–80% HRR), ST (RPE 14–17); **Alternative**: Self-selected intensity (suitable for those uncomfortable with HR monitoring).	Use RPE scale as key basis if HR monitoring unavailable; self-selected intensity group requires periodic HR check (ensure equivalence to standard intensity).	Avoid relying solely on subjective feeling; combine objective indicators (e.g., walking speed and HR); decrease intensity by 10–20% for significant non-motor symptoms.	Alberts and Rosenfeldt, 2020; Kanegusuku et al., 2021
Exercise type	Motor symptoms (targeted enhancement)	**Cardiopulmonary capacity**: brisk walking, swimming, cycling (AE); **Muscle strength/balance**: unstable ST, Tai Chi, pilates; **Gait**: complex gait training and dance.	Combined training (AE + RT + balance) superior to single type; prioritize Tai Chi for balance training (highest evidence level); include 5-min warm-up + 5-min cool down per session.	Exercise type must match patient's exercise capacity (e.g., avoid swimming in late stage and prevent drowning).	Zhou et al., 2022; Zhen et al., 2022; Kim et al., 2025; Liu et al., 2019; Gao et al., 2014; Çoban et al., 2021
Frequency & duration	Motor/non-motor symptoms (common)	**Early-mid PD**: 3–5 sessions/week, 30–60 min/session; **Late PD**: 2–3 sessions/week, 20–30 min/session (can be split into two segments).	Long-term intervention (≥6 months) requires “maintenance phase” (e.g., initial 3 weeks high intensity then switch to 常规强度); late stage duration should not exceed 30 min/session to prevent fatigue; reference ACSM standards: beginners 2–3 sessions/week, advanced 4–5 sessions/week.	Frequency should consider patient's lifestyle (e.g., 3 times/week easier to adhere); if single session impossible in late stage, split into “15 min × 2 sessions”/day.	Griffith et al., 2025; Rosenfeldt et al., 2021; Tollár et al., 2019; American College of Sports Medicine, 2009
Progressive Principle	Motor/non-motor symptoms (common)	**Initial phase**: Low intensity (50–60% HRmax), short duration (15–20 min), simple movements; **Adjustment phase**: increase intensity by 5–10% or duration by 5–10 min every 2 weeks.	Adjustment basis: no extreme fatigue or symptom lowering 24 h post-exercise; if discomfort occurs, immediately revert to previous phase parameters.	Avoid “rapid progression,” particularly in mid-late stage patients; monitor tolerance for 3–5 days after each adjustment.	Elbers et al., 2015
Personalized synergistic approaches with medication					
Synergy with key medications	Motor symptoms (enhanced drug efficacy)	**With levodopa**: voluntary wheel running (animal studies), regular AE (clinical); **With dopamine agonists**: AE + RT; **With anticholinergics**: Tai Chi, yoga (mind–body training).	**Levodopa**: exercise should coincide with drug “ON” period to increase efficacy; **Agonists**: exercise may decrease drug dose requirement (reducing side effects, such as sleepiness).	Assess drug side effects before combination (e.g., avoid exercising alone if sleepy from agonists); avoid high-intensity exercise during levodopa “OFF” periods.	Aguiar et al., 2013; Montgomery et al., 1994; Hristova and Koller, 2000; Tarsy, 2006
Management of drug side effects	Non-motor symptoms (drug side effects)	**LID**: basal ganglia regulation exercises (e.g., precise gait training); **Orthostatic hypotension (OH)**: AE + postural training (sit-to-stand step practice).	**LID**: exercise frequency 3 sessions/week, 30 min/session, focus on “low-impact + precision control”; **OH**: postural training daily 1–2 times, 10 min/session.	LID patients avoid high-intensity exercise to prevent symptom worsening; OH training requires BP monitoring, avoid sudden position changes.	Aguiar et al., 2013; Fanciulli et al., 2020
Dynamic adjustment by medication window	Motor symptoms (adapted to medication window)	**“ON” period (drug effective)**: moderate- to high-intensity exercise (HIIT, complex balance training); **“OFF” period (drug ineffective)**: low-intensity exercise (seated stretching, breathing exercises); **Wearing-off**: key training when drug concentration peaks.	**“ON” period goal**: maximize neuroplasticity (e.g., HIIT 2 times/week); **“OFF” period goal**: maintain basic capacity, not pursue enhancement.	Record “medication window” with patient, match exercise timing; avoid complex movements during “OFF” period to prevent falls.	Milani et al., 2024

AE, aerobic exercise; ST, strength training; VO_2_peak, peak oxygen uptake; H&Y stage, Hoehn & Yahr stage; RPE, rating of perceived exertion; HRmax, maximum heart rate; HRR, heart rate reserve; ACSM, American College of Sports Medicine; RT, resistance training; LID, levodopa-driven dyskinesia; OH, orthostatic hypotension; HIIT, high-intensity interval training.

The biomarkers listed in this table differ substantially in clinical readiness. BDNF and inflammatory markers are relatively feasible for repeated peripheral measurement, but their relationship with clinically meaningful improvement remains inconsistent. Oxidative stress markers may be sensitive to acute exercise load, diet, medication, and comorbidities. Neuroimaging markers provide more direct information on brain network function but are costly and not yet practical for routine exercise prescription. Therefore, these biomarkers should be interpreted as promising research tools rather than validated clinical indicators for individualized exercise dosing.

#### 3.5.1 Personalized exercise intensity

Developing a personalized exercise intensity plan is a critical step to ensure that PD patients benefit safely and effectively from exercise intervention. This requires moving beyond a “one-size-fits-all” model and comprehensively assessing the patient's exercise capacity, disease severity, non-motor symptoms, and quality-of-life needs to formulate a highly targeted exercise prescription.

PD patients display significant individual differences in exercise capacity, which decreases with disease progression, making it a main consideration for identifying exercise intensity. For early-stage PD patients, whose exercise capacity is comparatively maintained, moderate- to high-intensity exercise is suitable. Literature confirms that such exercise can induce neuroplastic changes. For example, one study, by comparing early PD patients with Pink1 gene knockout rats, established equivalent AE parameters, pointing out that during a non-contact boxing program, heart rate increased by about 35% in early PD patients, with significant enhancements in the “Timed Up and Go” test and cognitive function tests (Salvatore et al., 2022). Another 6-month study further pointed out that high-intensity endurance exercise significantly increased VO_2_peak in early PD patients, facilitating their motor capacity, and these advantages were not affected by autonomic dysfunction (Griffith et al., 2025). For mid-to late-stage patients, because of severely limited exercise capacity, enhanced fatigue, and high fall risk, exercise intensity should focus on low-intensity, low-impact activities. Gentle stretching exercises and Tai Chi are ideal choices. A systematic review determined that stretching exercises effectively impact motor symptoms and functional activities (Duñabeitia et al., 2025). Tai Chi, as a mind–body exercise, characterized by slow, flowing movements, aids in enhancing sensorimotor combination, decreasing force variability, and enhancing balance, making it notably suitable for patients with poor exercise capacity (Kerr et al., 2016).

Disease severity, generally assessed using tools such as the Hoehn & Yahr staging, directly determines the focus and form of exercise intervention. In the early stages of the disease (H&Y 1–2), patients can better tolerate and participate in comprehensive training programs. Studies show that adding 12 weeks of AE or RT to standard care significantly enhances patients' VO_2_peak and muscle strength, and exercise intensity can be safely and progressively increased (Demonceau et al., 2017). For patients with more severe or late-stage disease (H&Y stages 3–4), the goal of exercise should shift to maintaining current functional status and preventing complications (e.g., falls), thus requiring careful control of exercise intensity. Literature finds that for late-stage patients, low- or medium-frequency treadmill training is more effective than high-frequency training in facilitating gait and reducing fall risk, implying that excessively high frequency may cause fatigue that counteracts benefits (Pelosin et al., 2017). At this point, the exercise plan should be more targeted; for example, balance training (e.g., slackline training) has been proven effective for facilitating postural instability and freezing of gait (Santos et al., 2017). Even low-impact whole-body activities, such as golf, are considered helpful for improving balance and postural control (Bliss and Church, 2021).

Non-motor symptoms in PD, such as depression, anxiety, cognitive dysfunction, and sleep problems, not only impact the patient's quality of life but also directly influence their willingness to adhere to exercise. Hence, personalized plans must incorporate these factors. External stressful events (e.g., the COVID-19 pandemic) can exacerbate psychological distress, leading to decreased physical activity and thereby worsening overall PD symptoms (e.g., rigidity, fatigue, and tremor; Van Der Heide et al., 2020). This confirms that for patients with unstable emotional states (e.g., anxiety and depression), prescribing high-intensity exercise may be counterproductive. In such cases, light AE that can decrease stress and promote wellbeing, such as walking in a supportive environment, aquatic exercise, or Tai Chi, should be prioritized. These exercises are of moderate intensity and can help patients build confidence and progressively increase activity levels.

The success of exercise intervention depends on whether it meets the patient's personal goals and needs, thereby enhancing their overall quality of life. If the patient's main requirement is to improve motor capacity and enhance daily activity ability, then the plan should focus on the combination of AE and ST. Evidence reviews report that this combination effectively enhances mobility, posture, and balance (Kola and Subramanian, 2023). If the patient's main requirement is to improve mood, alleviate stress, or boost sleep quality, then the proportion of mind–body balance and relaxation training should be increased. Exercises such as Tai Chi and Yoga display unique advantages in this regard. A preliminary study reported that 16 weeks of Tai Chi exercise significantly increased the emotional wellbeing scores on the quality of life scale in PD patients, demonstrating beneficial effects on non-motor symptoms (Nocera et al., 2013).

Overall, the development of personalized exercise intensity is a dynamic, multi-factor decision-making process. It is not determined solely by objective exercise capacity tests; it also requires close communication between doctors and patients, integrating the patient's disease stage, emotional and cognitive state, and personal life goals to truly achieve patient-centered precision rehabilitation.

#### 3.5.2 Structured intervention protocol

Designing a personalized exercise intervention protocol requires translating exercise intensity into specific, quantifiable, and executable parameters. These encompass exercise mode, type, frequency, duration, and the principle of progression. Together, they form the core framework of a precise exercise prescription, ensuring that the intervention enhances physiological adaptation while being conducted safely within the patient's tolerable range.

Exercise intensity is the most critical factor determining the effectiveness of exercise interventions. For PD patients, the recommended intensity for AE generally ranges from 60% to 80% of the patient's maximum heart rate (HRmax) or 60% to 80% of heart rate reserve (HRR). This intensity range has been proven to effectively induce neuroplastic changes and enhance motor symptoms (Alberts and Rosenfeldt, 2020). When heart rate monitoring is unavailable, the Rating of Perceived Exertion (RPE) scale can be used, setting the intensity target at 14–17 points (corresponding to “somewhat hard” to “very hard”). Notably, allowing patients to self-select intensity is a viable approach to increase long-term adherence. Literature confirms that when PD patients are permitted to self-select AE intensity, their final heart rate levels, blood pressure responses, subjective fatigue, and emotional experiences display no significant differences in comparison to exercising at traditionally prescribed intensities (60–80% of HRmax; Kanegusuku et al., 2021). This offers a personalized and viable alternative for patients who find strict intensity monitoring stressful or uncomfortable.

The choice of exercise type should be based on the key symptoms of PD and individual patient goals. AE, such as brisk walking, running, cycling, and swimming, mainly aims to enhance cardiopulmonary endurance (VO_2_max) and overall motor capacity. A systematic review and meta-analysis further pointed out that AE significantly enhances patients' balance ability (Berg Balance Scale), gait speed, stride length, and overall motor capacity (assessed utilizing UPDRS-III; Zhen et al., 2022). Crucially, exercise dosage is significant: Combined training protocols integrating AE and other elements, as well as single-session durations ≥60 min, lead to greater improvement in motor symptoms than AE alone or shorter sessions (Kim et al., 2025). ST aims to counteract muscle atrophy and increase muscle strength, thereby improving gait stability and postural control. Literature shows that ST alone can effectively increase muscle strength, but if the goal is to simultaneously enhance balance, cognition, and quality of life, then RT integrated with instability training is recommended. For example, utilizing unstable surfaces, such as a BOSU ball during half-squat exercises, can increase the neuromuscular challenge of the movement, hence more effectively improving balance, fear of falling, and cognitive function (Silva-Batista et al., 2016). Moreover, a network meta-analysis determined that short-term high-intensity RT is notably effective in facilitating UPDRS-III scores (Zhou et al., 2022). Balance training is essential for fall prevention. Tai Chi is the most extensively studied balance training method with the highest level of evidence. Meta-analyses confirm that Tai Chi significantly reduces fall rates and promotes performance on the Berg Balance Scale, functional reach, and TUGT (Liu et al., 2019). RCTs also display that 12 weeks of Tai Chi exercise significantly enhances balance and effectively reduces fall frequency during a 6-month follow-up period (Gao et al., 2014). Besides, clinical Pilates has been proven to be an effective method for facilitating dynamic balance (Çoban et al., 2021).

For early to mid-stage PD patients, it is recommended to exercise 3–5 times per week, with each session lasting 30–60 min. This regular regimen has been reported to yield significant benefits. For example, 6 months of high-intensity or moderate-intensity endurance training, 3 times per week, significantly increased patients' VO_2_peak and promoted motor capacity, and these enhancements were not affected by baseline autonomic function status (Griffith et al., 2025). Eight weeks of high-intensity AE, 3 times per week, not only strengthened information processing speed and motor function but its effects also persisted for several weeks after cessation of exercise (Rosenfeldt et al., 2021). Long-term adherence is crucial; a 2-year study reported that initial high-intensity sensorimotor agility training for 3 weeks, followed by maintenance training 3 times per week, effectively delayed the development of PD symptoms (Tollár et al., 2019). For late-stage patients, because of more severe fatigue and movement limitations, exercise frequency can be appropriately altered, centering on low-intensity, low-risk activities, such as seated ST or gentle balance exercises, to avoid extreme fatigue. The American College of Sports Medicine (ACSM) reported a development model for ST in healthy adults, implying that beginners (untrained or not trained for years) should train 2–3 times per week; intermediate trainees (with about 6 months of consistent training experience) should train 3–4 times per week; advanced trainees (years of training experience) should train 4–5 times per week. These recommendations can help guide the frequency and intensity selection for late-stage patients during exercise (American College of Sports Medicine, 2009).

The implementation of the exercise plan must follow the principle of gradual progression. The initial phase should start with low intensity and short duration, allowing sufficient time for the patient to adapt. Subsequently, based on the patient's tolerance and feedback, exercise intensity, duration, or movement complexity should be progressively increased. Dynamic adjustment throughout the intervention process is key to ensuring safety and effectiveness. Therapists should closely monitor the patient's subjective fatigue, post-exercise reactions, and any discomfort symptoms. PD patients generally experience fatigue, a complex non-motor symptom the management of which requires comprehensive consideration (Elbers et al., 2015). If the patient experiences persistent severe fatigue or worsening of symptoms, the exercise intensity should be promptly decreased rather than mechanically adhering to the original plan. This flexible, patient-responsive adjustment approach is the key guarantee for the success of a personalized protocol.

#### 3.5.3 Combination of exercise and pharmacological therapy

The combined application of exercise and pharmacological therapy represents a significant development in PD management, shifting from passive symptom control to active, multimodal functional restoration. These two intervention approaches are not independent; rather, they generate significant synergistic effects, enhancing overall treatment effectiveness while effectively managing issues arising from long-term medication use.

Levodopa, the “gold standard” for PD treatment, effectively supplements brain dopamine and improves motor symptoms. Nevertheless, long-term use can cause motor complications, particularly LID. Combining exercise intervention with levodopa has been reported to increase effectiveness and mitigate these side effects. Animal experiments provide direct mechanistic evidence. Literature on a 6-hydroxydopamine-lesioned PD mouse model revealed that allowing mice to engage in voluntary wheel running while administering levodopa significantly prevented the development of LID (Aguiar et al., 2013). The molecular mechanism lies in the ability of exercise to normalize abnormal dopamine signaling in the striato-pallidal pathway, thereby suppressing the overexpression of aberrant signaling molecules, such as phosphorylated dopamine- and cAMP-driven phosphoprotein, 32 kDa (DARPP-32) and c-Fos. This confirms that exercise can not only enhance symptoms but also regulate the brain's response to medication at the pathophysiological level. At the clinical level, comprehensive patient education and health promotion programs (e.g., the PROPATH project) show that encouraging patients to engage in regular exercise integrated with optimized medical control brings numerous benefits. Patients engaging in such programs increased their physical activity, reduced “OFF” time and proportion, experienced fewer medication side effects, and reported approximately 10% enhancement in overall PD scores. More critically, the levodopa dose in the intervention group remained stable or even slightly decreased, while the control group's dose showed an upward trend (Montgomery et al., 1994). These findings suggest that exercise may complement pharmacological management and improve functional outcomes in some patients. However, current clinical evidence is insufficient to conclude that exercise reliably reduces medication requirements. Future trials should prospectively record levodopa equivalent daily dose, OFF time, dyskinesia severity, adverse events, and exercise adherence to clarify whether exercise can modify medication needs.

Besides levodopa, exercise can also interact effectively with dopamine agonists, anticholinergic drugs, etc. Although high-quality RCTs directly studying the synergy between exercise and these specific drugs are comparatively scarce, reasoning by pathophysiology and clinical observations supports their synergistic effects. For example, for patients utilizing dopamine agonists, exercise can enhance cardiopulmonary capacity and muscle strength, improving the patient's overall endurance and motor capacity. This may allow satisfactory effects with lower doses of agonists, hence reducing the risk of side effects, such as drowsiness and impulse control disorders (Hristova and Koller, 2000). For younger patients with predominant tremor utilizing anticholinergic drugs, exercise (especially mind–body practices, such as Tai Chi and Yoga) can assist in tremor control by enhancing sensorimotor combination and reducing anxiety levels, possibly reducing dependence on anticholinergic drugs and hence avoiding cognitive and autonomic side effects, such as memory dysfunction and constipation (Tarsy, 2006). Moreover, the benefits of exercise for mood, sleep, and pain can decrease the patient's need for related symptomatic medications (e.g., antidepressants, sleeping pills, and analgesics). For example, AE and ST can mitigate common musculoskeletal pain in PD through processes, such as enhancing muscle strength and endorphin release (Edinoff et al., 2020). This enhancement in non-motor symptoms reduces reliance on adjuvant medications, simplifies the treatment regimen, and can increase quality of life.

Exercise demonstrates unique value in managing drug side effects, particularly in dealing with LID and drug-driven autonomic dysfunction. As pointed out earlier, exercise can directly regulate basal ganglia circuit plasticity and is a promising non-pharmacological approach for preventing and decreasing LID. Besides, many PD patients suffer from orthostatic hypotension (OH), which is both a manifestation of the disease itself and can be exacerbated by dopaminergic drugs. Although pharmacological treatment (e.g., midodrine and fludrocortisone) is a significant approach for managing OH, non-pharmacological measures are the cornerstone (Fanciulli et al., 2020). Regular AE and ST can enhance cardiovascular regulation and increase vascular tone; targeted movement training (e.g., step-by-step sit-to-stand practice) can enhance hemodynamic responses during postural changes, thereby reducing the occurrence and severity of OH.

When developing personalized treatment plans, medications and exercise should be considered as an integrated whole for dynamic planning. The key lies in making precise modifications to the exercise prescription based on the patient's response to medication and side effects (Milani et al., 2024). For patients with good medication control and in the “ON” period, the window of better motor capacity should be completely utilized. At this time, moderate- to high-intensity, more complex exercises can be scheduled, such as HIIT, RT integrated with instability, dance, or complex gait training. The goal is to enhance the induction of neuroplasticity and functional enhancement (Milani et al., 2024). When the patient is in the “OFF” period or adjusting medication doses, the exercise plan should prioritize safety and tolerability. Low-intensity, low-impact activities should be chosen, such as seated exercises, gentle stretching, breathing exercises, or aquatic exercises. The focus is on maintaining joint range of motion, muscle flexibility, and basic cardiopulmonary capacity, avoiding abandonment of exercise because of extreme fatigue or fall risk (Milani et al., 2024). For patients experiencing wearing-off phenomena, attempt to schedule the main training when drug concentration peaks and motor capacity is optimal. Simultaneously, teach patients approaches for safe home activities during “OFF” periods (Milani et al., 2024).

### 3.6 . Future studies directions and challenges

#### 3.6.1 . Lack of quantification standards

One of the key bottlenecks in current PD exercise intensity literature is the absence of unified, scientific, and reproducible standards for quantifying effects. This hinders the cross-comparison of different study findings and can lead to inefficient clinical translation. Recent evidence confirms this issue manifests mainly in two dimensions: confusion in defining exercise parameters and heterogeneity in evaluation metrics. Regarding exercise parameters, significant discrepancies exist in how different studies define “intensity.” A series of studies use “percentage of HRmax” as a benchmark [e.g., moderate intensity defined as 70–80% of HRmax (Maass et al., 2015)], while others employ RPE (Silva-Batista et al., 2016). Several studies even vaguely describe intensity as “high” without providing specific quantitative criteria. Regarding exercise type and duration, operational definitions for “AE” and “RT” remain inconsistent (e.g., no standardized load weight or repetition counts for RT). Moreover, there is a lack of a unified reference framework for comparing the effectiveness of short-term interventions (8 weeks; Chang et al., 2018) vs. long-term interventions (12 weeks; Mak et al., 2017). Regarding evaluation metrics, recent studies predominantly employ single-dimensional indicators (e.g., utilizing only UPDRS-III to evaluate motor symptoms; Zhou et al., 2022) while neglecting the inclusion of non-motor symptoms (e.g., utilizing PSQI to assess sleep; Pezzini et al., 2024) and neurobiological markers (e.g., BDNF levels; Kaagman et al., 2024). This failure prevents comprehensive quantification of the multidimensional effects of exercise intensity.

To address this issue, future studies need to establish a quantification standard system from numerous aspects. First, operational definitions of exercise parameters should be unified. Based on recent evidence consensus, researchers should establish quantitative methods for key parameters of different exercise categories (AE, RT, Balance, HIIT). For example, AE intensity requires simultaneously reporting “percentage of max HR” and “percentage of VO_2_peak” [referencing Griffith et al.'s (2025) use of VO_2_peak to assess endurance training effects]; RT requires specifying details, such as “load weight (e.g., 50–70% 1-RM), repetitions (10–15/set), and rest interval between sets (60 seconds)” to ensure reproducibility across studies. Moreover, building a multi-dimensional evaluation indicator system is crucial, particularly mandatorily integrating three categories of indicators: “clinical symptoms—neural processes—quality of life.” Clinical symptoms need to cover motor symptoms (UPDRS-III) and non-motor symptoms (HAM-D for depression, MoCA for cognition, PDSS for sleep); neural mechanism indicators need to include BDNF [serum/CSF levels (Ruiz-González et al., 2021)] and inflammatory factors [IL-1β, TNF-α (Wang et al., 2021)]; quality of life indicators should use the PDQ-39 scale, avoiding the limitations of single indicators. Building stage-adapted standard thresholds is also needed, calibrating quantification standards by PD course (Hoehn-Yahr stage). For example, the high-intensity exercise standard for early-stage patients (H&Y 1–2) can be set as “80–85% max HR, 3 times/week, 45 min/session” [referencing Schenkman et al.'s (2018) HIIT protocol for newly diagnosed patients], while for late-stage patients (H&Y stages 3–4), it needs to be decreased to “50–60% max HR, 3 times/week, 30 min/session,” with “no fall risk” as the safety threshold, ensuring the clinical applicability of the standards.

#### 3.6.2 Long-term effect studies

Current evidence on PD exercise intensity mainly focuses on short-term interventions (8–12 weeks), which can demonstrate exercise's symptomatic advantages [e.g., 8 weeks of low-intensity cycling training decreased UPDRS-III scores (Chang et al., 2018)]. Nevertheless, the lack of quantitative analysis on long-term intervention effects (≥2 years) and disease progression prevents determining whether exercise has durable symptomatic benefits or potential disease-modifying effects (i.e., influencing disease trajectories rather than producing only short-term symptomatic improvement). Among the limited recent long-term studies, (Tollár et al. 2019) reported that 2 years of agility maintenance training delayed PD symptom development. However, the study had a small sample size (no confirmation offered) and could not quantify exercise's influence on key pathological processes, such as “dopaminergic neuron loss rate” or “Lewy body spread.” In addition, most evidence cannot track the “duration of effect maintenance” after exercise cessation—only one article published by (Rosenfeldt et al. 2021) has pointed out that the effects of 8 weeks of high-intensity AE could persist for several weeks. Nevertheless, the decay pattern of effects after long-term discontinuation remains unclear. Another limitation of recent long-term studies is the inability to distinguish between the synergistic and independent effects of exercise vs. medication. Most studies involve patients concurrently receiving levodopa therapy [e.g., (Aguiar et al. 2013) reported that integrated exercise and levodopa decreased dyskinesia]. Nevertheless, the studies failed to quantify whether exercise could decrease medication dosage or mitigate the onset of end-of-dose phenomena, hence precluding independent assessment of exercise's influence on disease progression.

Future studies on the long-term effects of exercise require the following approaches. It is crucial to design large-sample, multi-center, longitudinal follow-up studies. Prospective cohort studies of at least 5 years are needed, enrolling PD patients at different disease stages (early unmedicated, mid-stage medicated, late-stage with complications). These studies should utilize an “exercise group (standardized protocol) vs. control group (usual care)” design and regularly (every 6 months) analyze key indicators. Clinically, this involves quantifying the UPDRS total score and sub-scores (motor, non-motor), fall incidence, and medication dose changes. Pathologically, it involves quantifying the protective effect of exercise on dopaminergic neurons and pathological protein aggregation through neuroimaging [e.g., PET detecting striatal dopamine transporter (DAT) density (Wang et al., 2013)] and CSF α-Syn levels.

Moreover, building a quantitative “exercise-disease course” association model is required. This involves introducing indicators such as “symptom development rate” (e.g., the annual increase in UPDRS scores) and “disease milestone time” (e.g., time from H&Y stage 2 to 3, time to first fall). Linear regression can then be utilized to analyze the association between exercise parameters (intensity, frequency, total duration) and these indicators, identifying “how much exercise dose can mitigate the disease course by X years” [referencing (Mak et al. 2017) on constructing the association between long-term exercise and symptom control]. Besides, separating the independent effects of exercise and medication is needed. This can be achieved by incorporating “medication adjustment records” (dose, type, timing) into the study design, utilizing statistical methods (e.g., propensity score matching) to control for medication factors, or conducting RCTs of “exercise + medication” vs. “medication alone.” The goal is to quantify the decrease in drug dependence due to exercise (e.g., whether the exercise group uses 20% less levodopa than the control group) and demonstrate the impact on motor complications [e.g., LID; referencing the conclusions of Aguiar et al.'s (2013) animal experiment].

#### 3.6.3 Integrated application of biomarkers

Current studies of exercise intensity in PD have generally used single biomarkers, such as serum BDNF or selected inflammatory markers, rather than integrated multi-omics and imaging approaches (Salvatore et al., 2022; Wang et al., 2021). This limits the ability to connect patient phenotype, molecular response, brain network adaptation, and clinical outcomes. Three limitations are particularly important. First, genetic factors such as BDNF Val66Met polymorphism are rarely incorporated into exercise-response models (Romero Garavito et al., 2025). Second, metabolomic and proteomic responses to exercise remain insufficiently characterized in PD. Third, few studies link molecular changes with imaging markers and clinically meaningful outcomes such as MCID, fall reduction, daily activity, or quality of life. Future studies should integrate genomics, proteomics, metabolomics, peripheral biomarkers, neuroimaging, medication status, and symptom-specific outcomes to identify which patients are most likely to benefit from specific exercise intensities.

Future efforts should build a biomarker application system through “multi-omics combination + personalized evaluation.” Genomics can guide motor response prediction by conducting genome-wide association studies (GWAS) in PD patients to identify genetic loci linked to “motor sensitivity.” For example, researchers should validate whether the BDNF Val66Met polymorphism can impact the magnitude of exercise-driven BDNF upregulation [i.e., whether Val/Val genotype patients benefit more from exercise than Met carriers (Romero Garavito et al., 2025)]. An “exercise response prediction model” could then be built using these loci to pre-match patients with optimal exercise intensity (e.g., Met carriers may be better suited for moderate-intensity rather than high-intensity exercise). Besides, metabolomics can decipher exercise-driven pathways utilizing liquid chromatography-mass spectrometry (LC-MS) to detect modifications in serum/cerebrospinal fluid metabolites before and after exercise intervention, identifying key metabolic pathways regulated by exercise. For example, researchers could assess whether AE enhances motor symptoms through upregulation of dopamine precursors (e.g., tyrosine and levodopa) or can mitigate oxidative stress by regulating fatty acid metabolism (e.g., enhancing ketone body production; Tsai et al., 2025). These metabolites can serve as “early biomarkers of exercise efficacy” (e.g., whether elevated ketone bodies after 2 weeks of exercise predict decreased UPDRS scores at 8 weeks). Building a “multi-omics-imaging-clinical” combined evaluation model is crucial. This requires combining genomics (e.g., BDNF genotype), proteomics (BDNF, IL-1β), metabolomics (ketone bodies, MDA), and radiomics (striatal DAT density, motor cortex functional connectivity) data. Machine learning (e.g., random forest algorithms) can then construct “exercise effect prediction models.” For example, inputting a patient's genotype + post-exercise BDNF changes + DAT density changes could yield personalized outputs, such as “probability (%) of exercise facilitating this patient's bradykinesia” and “cognitive function maintenance duration (months),” achieving a shift from “empirical exercise prescriptions” to “precision exercise prescriptions” [referencing (Kaagman et al. 2024) meta-analysis on BDNF-clinical effect correlations].

## 4 Conclusion

This review summarizes current evidence on the role of exercise intensity in managing motor and non-motor symptoms in PD. Available evidence suggests that low-intensity exercise may be suitable for improving tolerance, mobility, safety, and adherence, particularly in frail patients or those with more advanced disease. Moderate-intensity exercise appears to offer a practical balance between safety and functional benefit for many patients with mild-to-moderate PD. High-intensity exercise, including HIIT, may improve cardiorespiratory fitness and selected motor outcomes in carefully screened and supervised patients, but its long-term safety, adherence, and superiority over moderate-intensity exercise remain uncertain. Exercise may influence PD-related symptoms through multiple biological pathways, including neurotrophic signaling, neuroplastic adaptation, oxidative stress regulation, inflammatory modulation, and changes in cerebral perfusion. However, the strength of evidence differs across mechanisms. Much of the mechanistic evidence remains preclinical or indirect, and peripheral biomarkers such as BDNF, inflammatory cytokines, oxidative stress markers, cortisol, α-synuclein species, and neuroimaging measures are not yet validated for routine clinical exercise prescription. Current evidence is limited by heterogeneous definitions of exercise intensity, small samples, short intervention periods, limited long-term follow-up, underrepresentation of advanced PD, and insufficient reporting of clinically meaningful outcomes such as MCID, fall reduction, real-world physical activity, medication changes, adverse events, and quality of life. Future studies should standardize intensity reporting, stratify participants by disease stage and risk profile, use symptom-specific exercise protocols for non-motor symptoms, and integrate clinical outcomes with validated biomarkers and neuroimaging measures. Exercise intensity should therefore be viewed not as a universal prescription, but as a modifiable parameter within individualized, supervised, and clinically grounded PD rehabilitation programs.
